# Terpyridine-based Pd(ii)/Ni(ii) organometallic framework nano-sheets supported on graphene oxide—investigating the fabrication, tuning of catalytic properties and synergetic effects†

**DOI:** 10.1039/d0ra02195d

**Published:** 2020-06-17

**Authors:** Ruirui Ren, Sa Bi, Linhong Wang, Wuduo Zhao, Donghui Wei, Tiesheng Li, Wenjian Xu, Minghua Liu, Yangjie Wu

**Affiliations:** College of Chemistry, Zhengzhou University Zhengzhou 450001 P. R. China; Henan Institute of Advanced Technology, Zhengzhou University Zhengzhou 450001 P. R. China; Beijing National Laboratory for Molecular Science, Institute of Chemistry, Chinese Academy of Sciences Zhongguancun North First Street 2 Beijing 100190 P. R. China

## Abstract

Tailoring the structures of catalysts and the arrangement of organic bimetallic catalysts are essential in both fundamental research and applications. However, they still impose enormous challenges such as size and active species distribution, ordered uniformity, and controllable composition, which are critical in determining their specific activities and efficiency. Herein, a novel terpyridine-based hetero-bimetallic Ni/Pd nanosheet supported on graphene oxide (denoted as GO@Tpy-Ni/Pd) was fabricated, which exhibited higher catalytic activity, substrate applicability and recyclability for the Suzuki coupling reaction under mild conditions. The catalytic mechanism was heterogeneous catalysis at the interface and the synergetic effect between Pd and Ni resulted in a little Ni(0)/Pd(0) cluster including Pd(ii)/Ni(ii) as a whole being formed through electron transfer on the catalytic surface. This phenomenon could be interpreted as the nanoscale clusters of Ni/Pd being the real active centre stabilized by the ligand and GO and the synergetic effect. The absorption and desorption of different substrates and products on Ni/Pd clusters, as calculated by DFT, was proved to be another key factor.

## Abstract 2

The synergistic effect between Ni and Pd atom was the crucial factor for enhancing catalytic activity.

## Graphical abstract

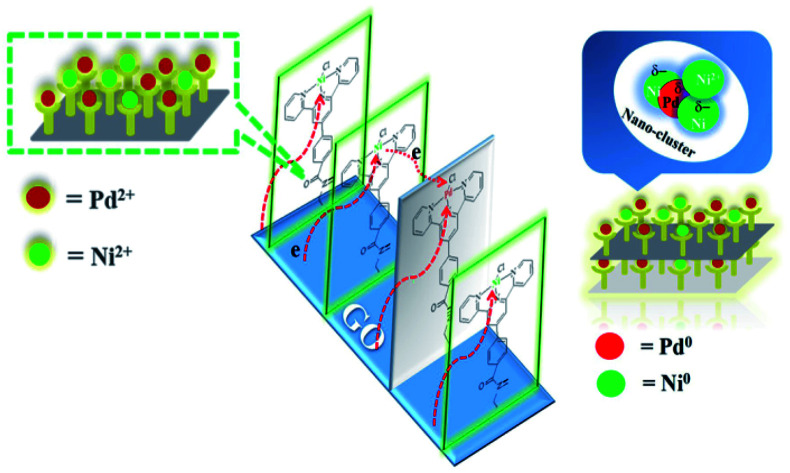

## 1. Introduction

Transition-metal-catalyzed cross-couplings have become truly fundamental tools for organic synthesis.^1–3^ Hetero-multi-metallic catalysts show new performances and capabilities due to the synergistic effects between the metals.^4–6^ In the last few decades, many hetero-bimetallic nanostructures, including nickel-based, nanoparticles, were widely used in industrial processes owing to their conspicuous activity and outstanding recyclability.^7–15^ These nanoparticles, with high catalytic activity, were also the most disposed to aggregation, which affected their catalytic performance.^16^ To solve this problem, appropriate supports and ligands, such as carbon materials and other materials, could strongly prevent metal species from aggregating.^17^ Nevertheless, practical supported hetero-multi-metallic nanocatalysts did not have well-distributed states that could reduce the catalytic efficiency and the emergence of undesired side reactions.^16^ It also made it extremely difficult to control the ratio and electron distribution, which were essential for enhancing catalytic activity.^18^ Therefore, understanding the fundamental mechanisms involved in how to form uniform active centres, why each component interacted and what was the optimized combination for deeply affecting the overall properties became more important research.^19,20^ It was also very important for the metal catalysts to be supported, since nano-particles of catalysts only having very weak interaction with supports might easily aggregate and leach, resulting in degrading performance. Covalent attachment was a very effective method for anchoring catalytic active centres.^21,22^ Therefore, the rational fabrication of the ordered self-assembled (SAM) catalytic monolayer and its functionality during catalysis must be investigated deeply,^23^ including how to identify every element, ensure the proper composition, and produce accurate and stable nano-catalysts.

Self-assembly (SAM) offers customized design, controllable orientation, ease of recovery and stable monolayers. It also has the advantages of homogeneity and heterogeneity through the ingenious design of single-molecular structures.^24^ Previous studies have shown that the catalytic activity of the hetero-bimetallic catalysts could be enhanced by tuning their composition, morphology and the relative distributions of electrons, which are related to the electrical characteristics of the ligand, supports, and the synergy of hetero-bimetals.^25–39^ Appropriate supports could promise a high dispersion of metallic catalyst and the maximum utilization of noble materials has attracted much attention;^19,40^ graphene oxide (GO) has been recognized as an ideal candidate for supporting various metals and their complexes.^41–43^ Pd catalyst doped with Ni, Fe, Co or Cu, reduces the loading of Pd and significantly enhances catalytic activity by adjusting the distance between nickel and palladium, charge distribution and the arrangement of active sites.^34,36,44^ Palladium was selected because of its efficient catalytic activity for Suzuki cross-coupling. However, its application was restricted by the limited reserves that were conventionally challenging for Pd catalysts.^2^ Nickel shares common chemical features and outer shell electron distribution with palladium.^45,46^ Cross-coupling reactions catalyzed by nickel have recently been attracting significant attention because of the lower toxicity, lower cost and greater nucleophilicity due to the smaller size of nickel as compared with Pd, which is critical for the Suzuki coupling reaction. Nickel cannot be simply considered to be a substitute for palladium since it possesses distinctive catalytic properties that palladium does not have.^47,48^ However, Ni is sensitive to solvent, a greater amount is needed, and severe catalytic conditions are usually required.^49–65^ The combination of the advantages of Pd and Ni for catalyzing the coupling reaction has recently attracted tremendous attention.^66^ The design of ordered, more cost-effective and eco-friendly forms to adjust catalytic properties and elucidate synergy is greatly needed.^67–69^

In this paper, the terpyridine group was selected as the binding site for palladium and nickel. The character of the terpyridine linkage with GO could provide the proper steric, coordination, and electronic requirements, leading to the bimetallic catalyst demonstrating high catalytic performance. The terpyridine Pd/Ni catalytic monolayer fixed on the graphene oxide nano-sheet was prepared and its catalytic performance and the synergistic effects between different metals were systematically investigated.

## 2. Experimental

### 2.1 Reagents and instruments and general procedure

Chemical reagents and instruments used for characterization, general synthesis and the general procedure for the coupling reaction and RactIR recording are provided in the ESI.†

### 2.2 Preparation and characterization of GO@Tpy-Ni/Pd nano-sheets

**Scheme 1:**
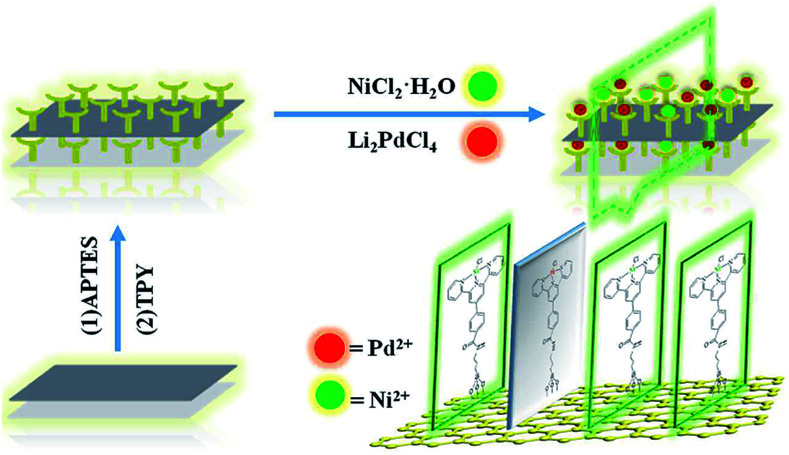
Preparation route of GO@Tpy-Pd/Ni nano-sheets.

The main target here was that the two different metals were arranged in monolayers, which were mutually improved to form higher active species that were different from both individuals. The designed bimetallic catalytic monolayer was fabricated as shown in Scheme 1. Characterization was as follows:

FT-IR spectra of GO, GO@APTES, GO@Tpy and GO@Tpy-Pd/Ni were obtained (Fig. S1†). Various peaks for C–O (1053 cm^−1^), C=O (1726 cm^−1^), C=C (161 cm^−1^), =C–H (1381 cm^−1^) and a broad peak from 3000 to 3500 cm^−1^ (–OH) revealed the existence of the carboxyl group, hydroxyl group, and epoxy group on GO.^70^ Compared with GO, the grafted ligand and organometallic compounds could be identified by observing the changes in the functional groups at different steps. After GO was modified with APTES, Si–O–C and Si–O–Si peaks appeared at 1026 cm^−1^ and 1110 cm^−1^, providing evidence of silylanization to give GO@APTES.^71^ The band for the stretching vibration of C=N was observed at 1614 cm^−1^, suggesting that terpyridine derivatives were linked with GO to yield GO@Tpy. The C=N peak in GO@Tpy was red shifted after coordinating with Ni/Pd solution, attributed to the electron delocalization of C=N due to coordination with metals to form GO@Tpy-Pd/Ni.

The intensity ratio of D/G in RS provides important information.^72^ The Raman spectra of GO, GO@APTES, GO@Tpy and GO@Tpy-Pd/Ni were measured (Fig. S2†). The *I*_D_/*I*_G_ ratio for GO was about 0.94, while those for GO@APTES, GO@Tpy and GO@Tpy-Pd/Ni were 0.98, 1.03 and 1.02, respectively. This suggested that more numerous but smaller sp^2^ carbon domains were introduced into GO and the graphene lattice became a little disordered after modification.^73^

GO, GO@APTES, GO@Tpy and GO@Tpy-Pd/Ni were also characterized by XRD (Fig. S3†). The XRD pattern of GO showed a peak at 10.4° due to the functional groups introduced.^74^ The XRD patterns of GO@APTES, GO@Tpy and GO@Tpy-Pd/Ni showed a broad peak at 2*θ* = 21°, confirming that the major oxygen-containing groups of GO were functionalized. Moreover, the diffraction peak at around 10.1° did not vanish, indicating that the structure of GO was not destroyed during the fabrication process.^75^

SEM and TEM images of GO, GO@APTES, GO@Tpy and GO@Tpy-Pd/Ni were obtained (Fig. S4†). It was apparent that the nano-sheets were layered structures that were closely associated with each other.^56^ The SEM images showed extending sheets of lateral dimensions ranging from a few to ten micrometers (Fig. S4A†). The SEM image of GO@APTES (Fig. S4B†), GO@Tpy (Fig. S4C†) and GO@Tpy-Pd/Ni (Fig. S4D†) also presented neat sheet-like structures, demonstrating that the ordered Pd/Ni monolayer was modified on GO. Moreover, other sheet-like structures were observed (Fig. S4E†). The TEM images of different steps were also measured (Fig. S4F–H†). The layer-like sheet of GO could also be observed, which was the evidence that the morphology of GO did not change during the modification steps. The catalysts having thin crumpled flakes with wrinkles and many folded regions made it easy for the substrate to access many active sites.

QCM is a useful method in on-line chemical and biological detection through monitoring the change in the mass on the surface of quartz, which also has the advantages of real-time online detection, high sensitivity, and easy operation.^76,77^ The detection principle is as follows:Δ*F* = −2*F*_0_2(*ρqμq*) − 1/2Δ*m*/*A*, −Δ*F* ∝ Δ*m*.

QCM was utilized to analyze the preparation process of Au@Tpy-Pd/Ni to simulate the preparation process of GO@Tpy-Pd/Ni. The results are listed in Table 1.

**Table 1:** Resonance frequency changes in the Au@Tpy-Pd/Ni nanosheet

Entry	Self-assembly operation	Frequency (*F*)/Hz	Δ*F*/Hz
1	Au	9 983 673	—
2	Au@OH	9 983 602	↓71
3	Au@APTES	9 981 510	↓2092
4	Au@Tpy	9 977 622	↓3888
5	Au@Tpy-Pd/Ni	9 974 864	↓2758

The frequency of the bare quartz wafer was 9 998 673 Hz. During the processes of silanization modification, ligand grafting and metal complexing, the frequency decreased by 71 Hz, 2092 Hz and 3888 Hz, 2758 Hz, respectively. The results illustrated that the quality of the quartz wafer increased during the self-assembly process, which also demonstrated that each self-assembly step on the quartz wafer went on.

The chemical elements in the preparation process of GO@Tpy-Pd/Ni were also measured by XPS analysis (Fig. S5†). The peaks of C 1s and O 1s were detected in GO. Si 2p, Si 2s, and N 1s were clearly detected after anchoring the terpyridine ligand in GO@Tpy. Compared with GO@Tpy, characteristic Pd 3d peaks at 337.92 eV and 343.22 eV denoted the bonding energy (BE) of Pd(ii) and the peaks at 855.27 eV, 861.62 eV, 873.42 eV, and 878.54 eV, assigned to the BE of Ni(ii), were detected for GO@Tpy-Pd/Ni (Fig. S6†). The characteristics presented above confirmed that the ordered self-assembly of Ni/Pd bimetallic catalytic nano-sheets was fabricated.

## 3. Results and discussion

### 3.1 Investigation of the catalytic properties of GO@Tpy-Pd/Ni nanosheets

#### 3.1.1 Evolution of the catalytic performance of GO@Tpy-Pd/Ni

The optimization conditions of Suzuki coupling reactions catalyzed by GO@Tpy-Pd_1_/Ni_1_ were initially carried out by screening various solvents, time, bases, temperatures, catalyst loading and substrate usage. The results are summarized in Table S1.† The best yield was obtained by using a H_2_O/EtOH mixture (v/v = 1 : 3) (entries 1–8). The influence of base was also examined, in which K_2_CO_3_ provided the best yield (95%) (entries 7 and 9–11). In addition, 35 °C and 8 h were determined to be the optimum conditions (entries 12–15). With different loadings of catalysts (1, 2 and 3 mg), 1 mg of GO@Tpy-Pd_1_/Ni_1_ was the optimized loading (entries 16–17) and the usage of substrate was examined, in which 4-bromotoluene (0.3 mmol) was the best loading (entries 18–19). Based on the results, the best catalytic conditions were K_2_CO_3_ as the base, the H_2_O/EtOH mixture (v/v = 1 : 3) as the solvent, GO@Tpy-Pd_1_/Ni_1_ catalyst (1 mg), 4-bromotoluene (0.35 mmol), 35 °C, and 8 h. These were used in further investigations.

#### 3.1.2 The effect of composition on the catalytic properties of GO@Tpy-Pd/Ni*_x_*

Employing optimized conditions, the effect of composition on the performance of GO@Tpy-Pd/Ni*_x_* was investigated and the results are listed in Table S2.† Among the three compositions tested, the catalytic yield of GO@Tpy-Ni was only as low as 17%. However, by doping palladium with nickel, the reactivity could be significantly improved, in which GO@Tpy-Pd_1_/Ni_10_ provided the best performance. Thus, it was likely that the proper ratio of palladium to nickel on the surface could effectively create a favourable micro-environment to promote catalytic activity. Also, doping with another metal exhibited electronic or steric effects that impacted its catalytic performance by a “cooperative effect”.^78^ This also implied that proper active sites on the surface could efficiently contact the substrates and catalyze the reaction.

#### 3.1.3 Substrate scoping in the Suzuki coupling reaction catalyzed by GO@Tpy-Pd_1_/Ni_10_

Under optimized conditions, Suzuki coupling reactions catalysed by *GO@Tpy-Pd_1_/Ni_10_* with various aryl halides and arylboronic acids were carried out. The results are depicted in Table S3.† The coupling reactions proceeded with electron-withdrawing and electron-donating aryl bromide and arylboronic acids to give biaryl products with high yields (Table S3,† entries 1–6). However, in the case of aryl chlorides, lower yields were obtained (entries 7 and 8). Coupling product could also be obtained in the case of benzyl bromide with naphthylboronic acid in high yield (entry 9), except with the thiophene derivative (entry 10) due to its poisoning properties toward active sites. These results showed that GO@Tpy-Pd_1_/Ni_10_ was an active Ni/Pd bimetallic catalyst for the synthesis of aromatic compounds with higher TON value.

#### 3.1.4 The influence of supports and functional structure on the catalytic performance

Comparison experiments were designed and carried out to investigate the effects of supports on catalytic properties (Table 2).

**Table 2:** Effects of supports on catalytic properties^a^

Catalyst	Pd loading (mol g^−1^)	Ni loading (mol g^−1^)	Molar ratio (Pd/Ni)	Yield^b^/%	TON
GO	—	—	—	0	0
NiCl_2_/Li_2_PdCl_4_	1.80 × 10^−5^	3.00 × 10^−5^	1 : 1.70	14	2333
Tpy modified Pd/Ni	3.92 × 10^−5^	6.89 × 10^−5^	1 : 1.76	31	2372
GO@Tpy-Pd	7.11 × 10^−5^	—	—	91	3839
GO@Tpy-Ni	—	3.28 × 10^−5^	—	17	1555
GO@Tpy-Pd_1_/Ni_10_	1.82 × 10^−5^	2.93 × 10^−5^	1 : 1.60	96	15 824
Gel@Tpy-Pd/Ni	1.68 × 10^−5^	2.81 × 10^−5^	1 : 1.67	59	10 535
Glass@Tpy-Pd/Ni	1.74 × 10^−5^	2.75 × 10^−5^	1 : 1.58	21	3620

a Reaction conditions: PhB(OH)_2_ (0.35 mmol), 4-bromotoluene (0.3 mmol), base (0.6 mmol), GO@Tpy-Pd_1−_*_x_*/Ni*_x_*: 1 mg solvent (5.0 mL) at 35 °C for 8 h.

b Isolated yield.

Coupling compounds were not detected by GO (entry 1). In the case of a simple mixture of Li_2_PdCl_4_ and NiCl_2_, only 14% yield was obtained (entry 2). When a mixture of ligand, Li_2_PdCl_4_ and NiCl_2_ without GO was used, 31% yield was obtained (entry 3). For GO@Tpy-Ni (entry 5), Gel@Tpy-Pd/Ni (entry 7) and Glass@Tpy-Pd/Ni (entry 8), lower yields were obtained, which showed poor activity as compared with GO@Tpy-Pd (entry 4) or GO@Tpy-Pd_1_/Ni_10_ (entry 6). GO@Tpy-Pd_1_/Ni_10_ also showed higher activity than GO@Tpy-Pd, although the Pd content of GO@Tpy-Pd_1_/Ni_10_ was one fourth that of GO@Tpy-Pd. It was evident that the Pd/Ni catalyst could enhance its activity due to the ordered orientation and synergy between nickel and palladium. The TOF value of GO@Tpy-Pd_1_/Ni_10_ was 5 times and 3 times greater than that of Glass@Tpy-Pd/Ni and Gel@Tpy-Pd/Ni (entry 7), respectively. This result clearly revealed that GO also had a great role in the activity.^79,80^ The role of GO containing the functional groups is to disperse and stabilize the bimetallic catalyst, which makes it more favorable for substrates to access the active center because of its two-dimensional configuration and the major interaction of the two metals. This led to efficient electron transfer between the supports, ligand and the catalytic active centre. The catalytic performances of GO@Tpy-Pd_1_/Ni_10_ were also compared with the literature (Table S4†).

#### 3.1.5 Stability and recycling experiment

**Fig. 1:**
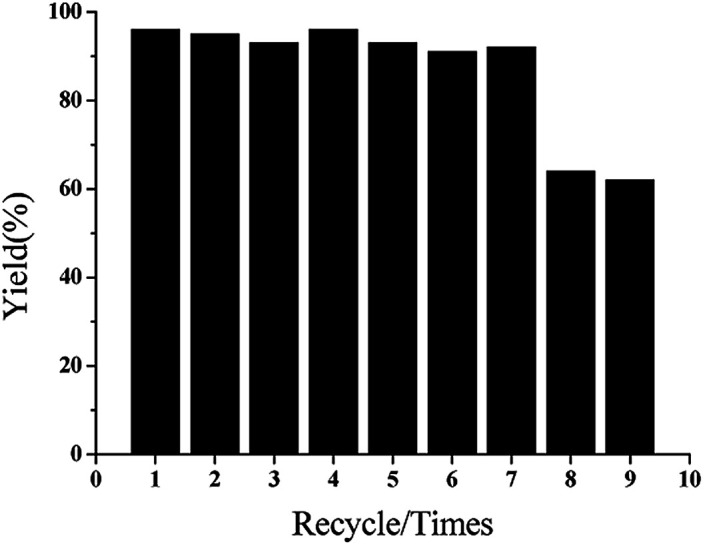
The recycling experiments of GO@Tpy-Pd_1_/Ni_10_ for the Suzuki coupling reaction.

To further investigate the recyclability of GO@Tpy-Pd_1_/Ni_10_, recycling tests were performed (Fig. 1). After the sixth run, high activity without a discernible loss could be maintained. However, lower yields were observed in seven cycles (61%). When the time was extended to 12 h in the eighth cycle, the yield increased to 85%. Unfortunately, in the ninth cycle, the yield again dropped to 64%, suggesting that the deactivation of the catalyst occurred in the catalytic process.^36–39^ In particular, when the freshly prepared GO@Tpy-Pd_1_/Ni_10_ was exposed for 1 month under ambient conditions, 90% yield could be obtained for the model reaction, indicating that GO@Tpy-Pd_1_/Ni_10_ was extremely stable.

**Fig. 2:**
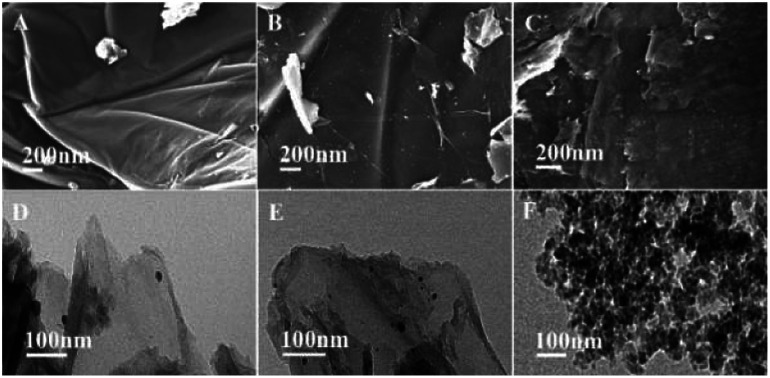
SEM and TEM images of GO@Tpy-Pd_1_/Ni_10_ (A and D) fresh catalyst, (B and E) after the 1st run, (C and F) after the 6th run.

To determine the decay mechanism, the changes in the catalyst during the catalytic process were characterized by TEM and SEM (Fig. 2). SEM and TEM after the 1st run showed no obvious changes in the surface morphology (Fig. 2B and E) as compared with the fresh catalyst (Fig. 2A and D). However, there was a clear accumulation on the catalytic surface after six cycles (Fig. 2C and F), which might be unavoidable for most supported metallic catalysts under such reaction conditions. Therefore, we speculated that the decrease in the activity might be due to the aggregation of active species, indicating that it was a structure-sensitive catalytic reaction.^81^ The Pd and Ni content in GO@Tpy-Pd_1_/Ni_10_ recycled for seventh was also measured, in which 1.34 × 10^−6^ mol g^−1^ and 1.48 × 10^−7^ mol g^−1^ were determined for palladium and nickel, respectively. It indicated that the leaching of metals was also one of the reasons for loss activity.

### 3.2 Investigation of the catalytic mechanism

#### 3.2.1 Hot filtration experiment

**Fig. 3:**
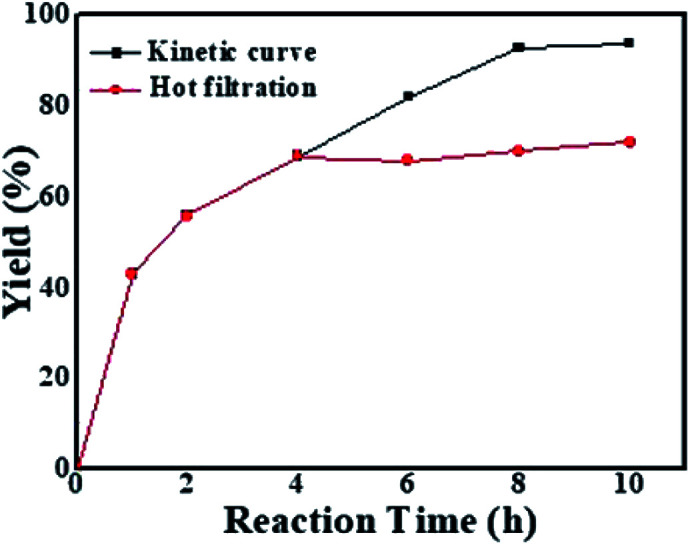
Kinetic curve and hot filtration experiment for GO@Tpy-Pd_1_/Ni_10_.

**Fig. 4:**
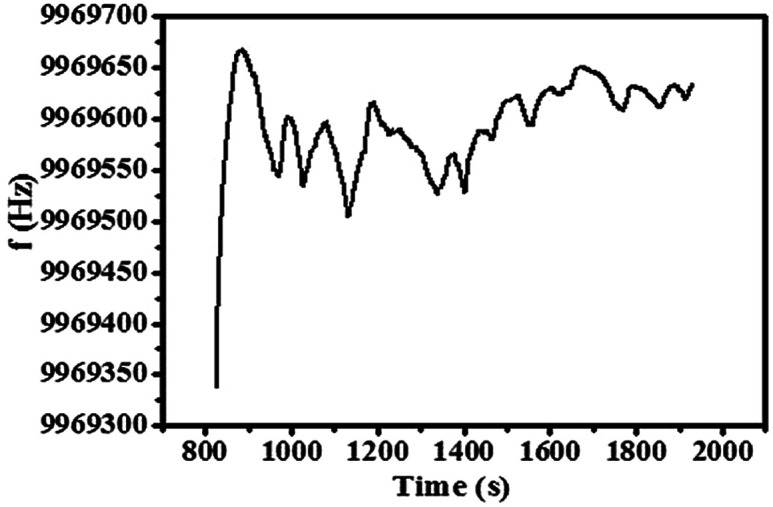
QCM responses of the sensor in the Suzuki reaction process catalyzed by Au@Tpy-Pd1/Ni10.

It is well known that it is important to distinguish heterogeneous catalysts from homogeneous catalysts. The yield increased rapidly before 2 h and then increased slowly after 2 h (Fig. 3, black line). The reaction was finished in 8 h with a 93% yield. The high activity of the GO@Tpy-Pd_1_/Ni_10_ was attributed to the favorable configuration and dispersion ability. In order to explore whether Pd leaching occurred during the catalytic process, GO@Tpy-Pd_1_/Ni_10_ was removed from the solution in 4 h and the yields were monitored. The yield showed no increase, remaining almost constant (69–71%, Fig. 4, red line), which indicated that no Pd leaching into solution occurred during the catalytic reaction process.^35,38^

#### 3.2.2 Poisoning tests

To elucidate the sites on which the catalysis proceeded, poisoning tests were designed and the results are summarized in Table S5.† When a little mercury was added to the catalytic system, only 14% yield was obtained because the limited active centres on the surface of the catalytic nano-sheet could be partially covered by strong poisons in sub-stoichiometric amounts of Hg due to the poor dispersibility of mercury. When thiophene additives were used, even with less than 1.0 equivalent, significant loss of activity was noted due to the strong chemisorption of thiophene on catalytic centres, thus blocking sites for catalytic reaction.^82^ The results provided evidence that the catalysis mainly proceeded on the surface of GO@Tpy-Pd_1_/Ni_10_.

#### 3.2.3 Quartz crystal microbalance (QCM) monitoring

Quartz crystal microbalance (QCM) has the advantages of real-time online detection, high sensitivity, and easy operation.^76,77^ For further insight into the catalytic mechanism, the Au@Tpy-Pd_1_/Ni_10_ self-assembled monolayer was fabricated on the quartz wafer with which the frequency change was detected during the catalysis. As shown in Fig. 4, the frequency of Au@Tpy-Pd_1_/Ni_10_ showed the “decrease and increase”. The change in frequency within 700–1300 s was more significant than that during 1300–1700 s. Correspondingly, the quality change on the quartz presented the “increase and decrease” in the catalytic process. This phenomenon indicated that adsorption and desorption occurred. It was clear that the rate of adsorption in the first half was greater than that of desorption, and the desorption rate in the second half was greater than that of adsorption. Finally, the adsorption and desorption reached an equilibrium. The results clearly showed the changes during the catalytic process, which indicated the absorption and desorption processes.

#### 3.2.4 *In situ* Fourier transform infrared (FTIR) spectroscopy monitoring

**Fig. 5:**
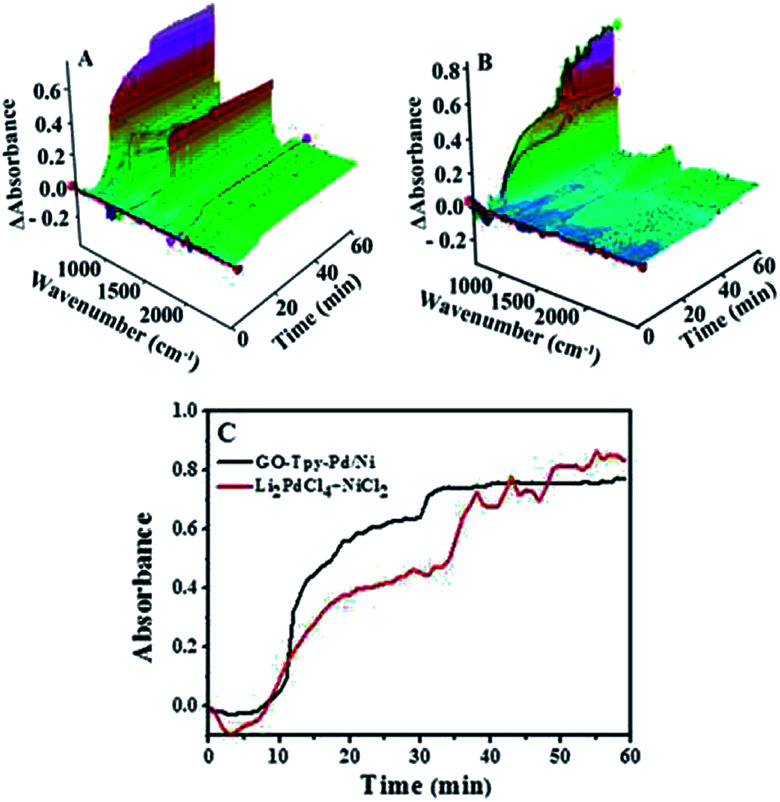
ReactIR plots over time for the formation of 4-phenyltoluene: (A) 3D map of by GO@Tpy-Pd_1_/Ni_10_; (B) 3D map of Li_2_PdCl_4_/NiCl_2_·6H_2_O; (C) kinetic analysis of the reaction catalyzed by GO@Tpy-Pd_1_/Ni_10_ and Li_2_PdCl_4_/NiCl_2_·6H_2_O using the band at 754 cm^−1^. Reaction conditions: PhB(OH)_2_ (1 mmol), 4-bromotoluene (1.5 mmol), base (2 mmol), solvent (6 mL), 35 °C, 1 h.

The ReactIR 3D maps over time by GO@Tpy-Pd1/Ni10 (Fig. 5A) and Li_2_PdCl_4_/NiCl_2_·6H_2_O (Fig. 5B) showed a marked difference. For GO@Tpy-Pd1/Ni10, the peak intensity at 754 cm^−1^ assigned to the coupling product, had no significant change from 0 to 10 min and increased rapidly with time. The final kinetic curve was “S”-shaped until the reaction was completed, which indicated the characteristic heterogeneous catalysis (Fig. 5C, black line).^36,83^ This could be because the surface of the catalytic monolayer contained a limited number of active centres. The substrates were first adsorbed on the surface without any reaction, which was called an “induced period”. Then, the intermediates were generated by reacting the substrate with specific active sites. Finally, the products were formed and diffused to the reaction solution from the catalytic surface. This was consistent with QCM detection.

When the same amount of Li_2_PdCl_4_/NiCl_2_·6H_2_O was used, no coupling product was observed because of the limitation of the amount of catalyst, which indicated that the orientation and distribution of active centres were crucial factors in catalysis. When a greater amount was added, the peak intensity of the product gradually increased within 10–35 minutes and rapidly increased until it reached equilibrium, in which the trend showed a “step” shape (Fig. 5C, red line); however, its intensity was lower than that of GO@Tpy-Pd_1_/Ni_10_. This provided evidence that the ordered bimetallic monolayer had a higher catalytic activity under the same conditions.

#### 3.2.5. SEM and TEM images of GO@Tpy-Pd1/Ni10 in the catalytic process

**Fig. 6:**
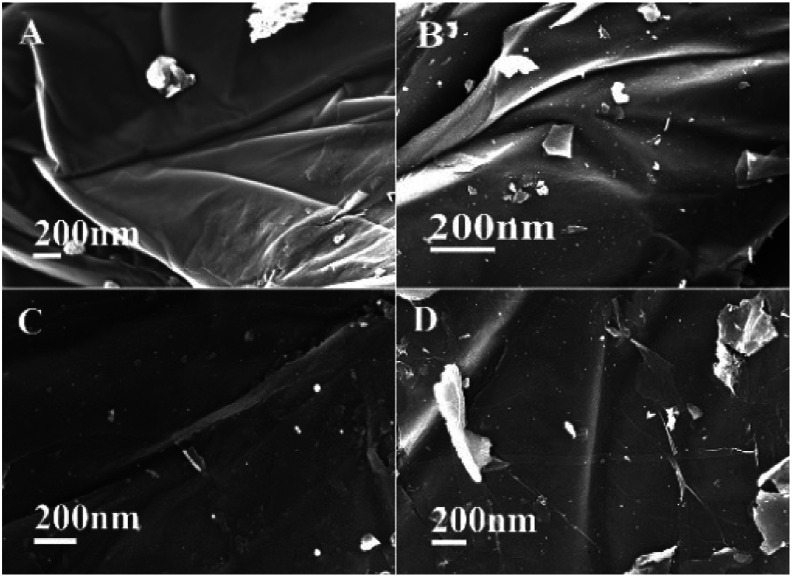
SEM images of GO@Tpy-Pd_1_/Ni_10_ at (A) 0 h, (B) 2 h, (C) 4 h, and (D) 8 h.

The surface morphology changes in the catalyst during the catalytic process were characterized by SEM and TEM. Compared with the GO@Tpy-Pd_1_/Ni_10_, the surface was still a lamellar folded structure after 2 h, 4 h, and 8 h and remained in the basic morphology as shown in Fig. 6. It could be concluded that the designed GO@Tpy-Pd_1_/Ni_10_ was structurally stable during the catalytic process.

**Fig. 7:**
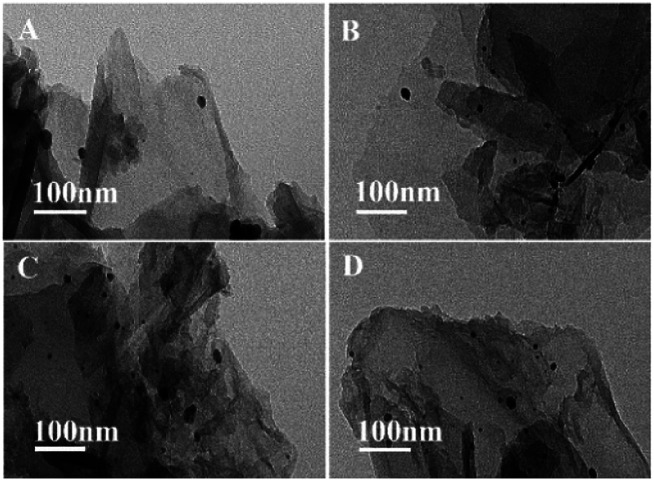
TEM images of GO@Tpy-Pd_1_/Ni_10_ for catalysis at (A) 0 h, (B) 2 h, (C) 4 h, (D) 8 h.

Correspondingly, TEM images of GO@Tpy-Pd_1_/Ni_10_ show a wrinkle-like two-dimensional thin-layer structure (Fig. 7) and nano-sized particles were generated due to the formation of palladium–nickel metal elements after 2 hours and 4 hours. This could be related to the aggregation of the catalytic species to form Ni/Pd clusters due to the migration of active atoms by coupling with each other through complex physicochemical processes during catalysis.^82^

#### 3.2.6 XPS investigation of GO@Tpy-Pd1/Ni10 in the catalytic process

**Fig. 8:**
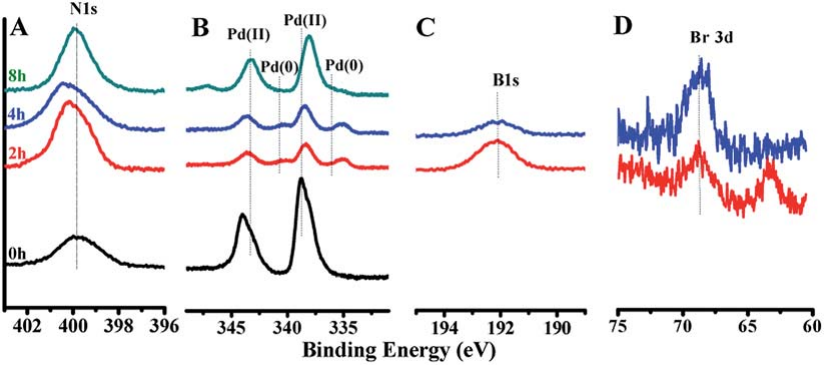
XPS of (A) N 1s, (B) Pd 3d, (C) B 1s and (D) Br 3d at 0 h, 2 h, 4 h, and 8 h.

The XPS survey spectrum and N 1s, Pd 3d, B 1s and Br 3d XPS spectra of GO@Tpy-Pd_1_/Ni_10_ during the catalytic process were obtained (Fig. 8). For the GO@Tpy-Pd_1_/Ni_10_, a pair of Pd 3d_3/2_ and Pd 3d_5/2_ peaks appeared at 340.74 eV and 335.14 eV, assigned to Pd(0) after 2 h (Fig. 8B), and the intensity of Pd(0) gradually increased with time. In contrast, the intensity of Pd(ii) gradually decreased during the catalytic procedure, meaning that a little Pd(ii) was reduced to Pd(0), which was the real active centre. The intensity of Pd(0) almost disappeared at the end, and the intensity of the Pd(ii) peak gradually recovered, suggesting that Pd(0) might be oxidized to Pd(ii). During this process, the N 1s energy level peak had also undergone certain changes (Fig. 8A), in which the N 1s peak appeared at 399.6 eV before catalysis. However, the N 1s peaks shifted to 400.29 eV (1 h), 400.44 eV (4 h) and 399.98 eV (8 h) during the reaction. It was not difficult to find that the position of the N 1s peak in the catalytic process showed a trend of first increasing and then decreasing. This was due to the electron transfer caused by the change in the valence state during the reaction between palladium and nickel that were coordinated with metals. Interestingly, B 1s and Br 3d were detected at 1 h and 4 h, but no B 1s and Br 3d at the end (Fig. 8C and D). Metal elements were generated after starting catalysis and the oxidation of benzyl bromide with metal to give intermediate Br–Pd–Ph inside or on the surface. Afterwards, reduced elimination was achieved by phenylboronic acid with intermediates to yield the coupling product on removing B and Br from the surface.

**Fig. 9:**
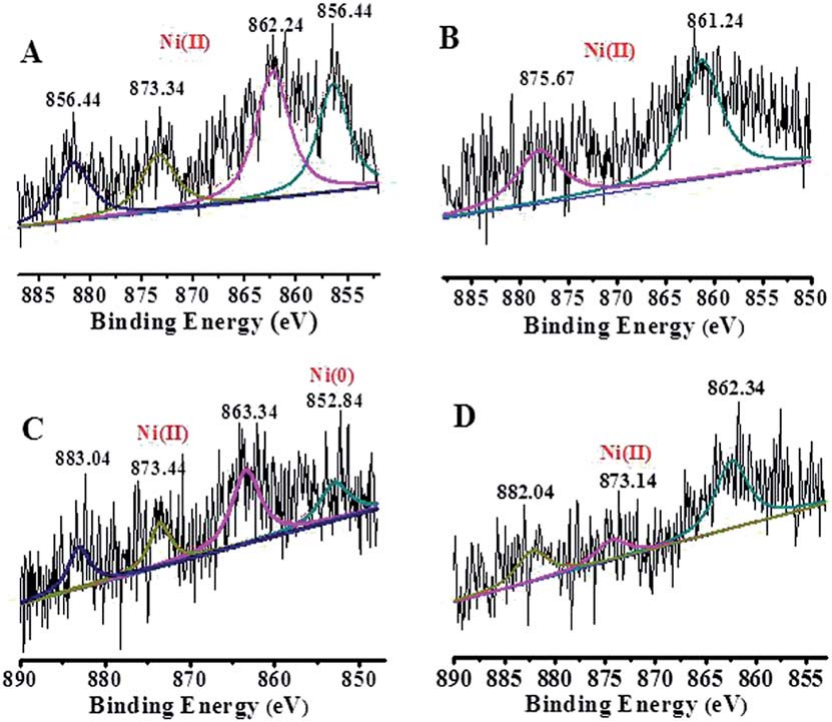
High-resolution XPS of Ni 2p at different times: (A) 0 h, (B) 2 h, (C) 4 h, and (D) 8 h.

**Scheme 2:**
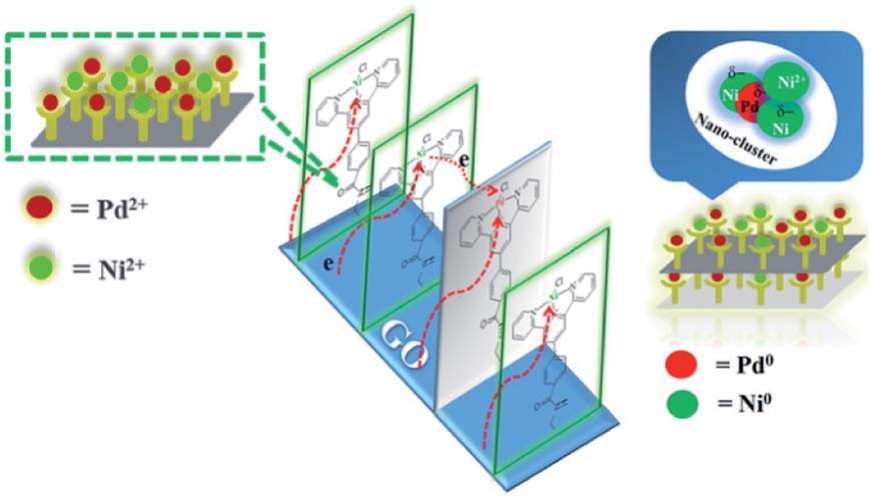
Proposed formation of the active centre Ni/Pd cluster during the catalytic process.

The energy level change in Ni 2p during the catalytic process was also analyzed (Fig. 9) and the binding energy of the Ni 2p energy peak was reduced after 2 h. The Ni 2p_3/2_ energy level peak and its satellite peak appeared at 852.84 eV and 863.34 eV after 4 h, which was attributed to Ni(0) being generated due to electron transfer from GO. This could be in the form of several atoms-clusters Ni/Pd during this time, which made it easy for the oxidation step because of much more negative active centres (Scheme 2). The binding energy of the Ni 2p energy level peak gradually returned to the pre-reaction state after 8 h and Ni(0) was oxidized to Ni(ii). Regarding the XPS spectra of Ni 2p and Pd 3d, the atomic cluster of Ni(0)/Pd(0) surrounded by appropriate Pd(ii)/Ni(ii) played a significant role in the catalysis due the synergistic effect, being mainly responsible for its higher activity. It was clear that the clusters were coordinated with ligand mostly at the surface of the organometallic framework. The surface can be regarded as an infinite “organometallic pool” containing a few active species,^84^ showing the great impact of the modification of a metallic surface by organometallic compounds on its catalytic properties.

The activity of the catalyst depended on the real active sites on the catalytic surface which were affected by the structure of the ligand and possibilities of electron transfer. In this catalytic monolayer, graphene oxide sheet itself showed some major possibilities of electron transfer and promoted the catalytic activity. The different possibilities of electron transfer in GO@Tpy-Pd_1_/Ni_10_ were as follows: (i) Ni(ii) to adjacent Pd(ii); (ii) Ni(ii) passed through adjacent graphene nanolayers to adjacent Pd(ii); (iii) Pd(ii), Ni(ii) to the adjacent carbon atom/graphene nano-sheet. The number of different ways for electron transfer depicted in GO@Tpy-Pd_1_/Ni_10_ was considerably higher as compared to the mono-metallic GO@Tpy-Pd and Si@Tpy-Pd_1_/Ni_1_ catalyst.

In order to further investigate the synergy between palladium and nickel presented during the catalytic reaction, density functional theory was utilized to calculate the energy barrier of the oxidative insertion process catalyzed by Pd(0) and Ni(0). The Pd–Br, Pd–C and C–Br distances were 3.13, 2.00, and 2.26 Å in transition state TS1, and the energy barrier of the oxidative insertion was 3.6 kcal mol^−1^ (Fig. S7†). The Ni–Br, Ni–C and C–Br distances were 2.84, 2.54 and 2.12 Å in transition state TS2, and the EB of the oxidative insertion was 17.8 kcal mol^−1^, which was higher than that of Pd(0) (Fig. S8†). The results showed that both Pd(0) and Ni(0) could form oxidation intermediates, although Pd(0) was much more active than Ni(0) in the oxidation step. The results were consistent with the experiments listed in Table 2.

One of the necessary steps during the catalytic reaction was the adsorption of one or more substrates. This was necessary for investigating the effects of the adsorbed substrates on the active species because it could help to elucidate the mechanism. The overall thinking was that the significant catalysts should integrate with substrates, intermediates or target molecules in certain concentrations to indicate whether the interaction force was too weak to activate the reactants and too strong to release the compounds.^85^ Thus, it provided a simple way to find the right metal combination in which different metals had various selectivities and functions, although many aspects must be simultaneously taken into account.^86–88^ DFT was used for investigating the absorption ability of the substrate and desorption ability of the product on different sites on the catalytic surface (Fig. S9†), by which the suitable selection of the bimetallic combination could be predicted.^89–92^ The results are shown in Table S6,† which were calculated by DFT (Fig. S10†).

**Scheme 3:**
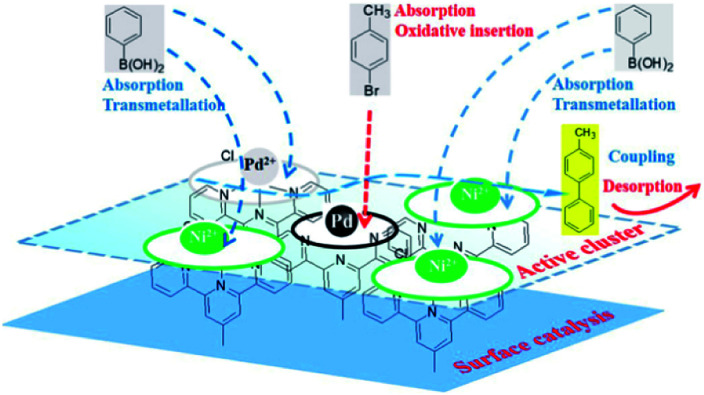
Synergy among the active sites during the catalytic process in the self-assembly of the organometallic framework films (OMFFs).

Pd(0) had a higher absorption for *p*-bromotoluene and a weakened absorption for phenyl boronic acid as compared to Pd(ii) and Ni(ii), indicating that the oxidative insertion could be easily completed by Pd(0) with *p*-bromotoluene. On the other hand, Pd(ii) or Ni(ii) in the vicinity of Pd(0) had a higher absorption ability for phenyl boronic acid, which facilitated metallic transfer, with the oxidative insertion intermediate formed on the next-nearest neighbor Pd(0) site. Pd(ii), Ni(ii) and Pd(0) had similar absorption abilities for coupling compounds. The DFT results obtained above showed that Pd(0) presented the higher activity because of its higher activation toward *p*-bromotoluene, and weaker absorption for the products. Meanwhile, Pd(ii) and Ni(ii) showed their stronger absorption for phenyl boronic acid, which was helpful for metallic transfer, and lower absorption for the coupling compound, which diffused easily from the surface. It was evident that the bimetallic catalyst had higher activity than the single metallic catalyst due to their synergy in position, orientation or distribution-selective processes, indicating that both active-sites and non-active sites participated simultaneously in the catalytic process as shown in Scheme 3. The presumed processes confirmed by DFT were consistent with the XPS variation during the catalytic process as shown in Fig. 9. We propose that new adsorption sites were likely formed with different electron densities, strengthening *p*-bromotoluene adsorption for Pd/Ni, which made Pd(0) compete more efficiently for adsorption sites in the form of the co-adsorption of phenyl boron acid and *p*-bromotoluene adsorption; therefore, nickel was considered as a promoter.^93^ This also suggests that Pd(0)/Ni(0) and Pd(ii)/Ni(ii) are not mutually exclusive, but have synergistic interactions as whole, indicating that one can consider that active clusters adopt a coordination sphere already present in the organometallic catalyst surface.^19^ This could be called the self-assembly organometallic framework nano-sheet (OMFNS), in which the morphology of the organometallic surface, orientation of the organometallic framework in the monolayer, kinds of ligands, support selected, ratio of bimetal, and interaction between aryl groups were crucial to the activity and stability of the catalyst.

## 4. Conclusion

A new graphene oxide-supported Ni/Pd terpyridine self-assembled catalytic monolayer (denoted as GO@Tpy-Ni/Pd) was fabricated, which exhibited higher catalytic activity, substrate applicability and recyclability as a heterogeneous catalyst for the Suzuki coupling reaction under mild conditions. QCM was used for characterizing and monitoring the reaction by the on-line detection of the frequency changes in a quartz wafer linked to the bimetallic catalyst, and clearly different rates of adsorption and desorption were presented during the catalytic process. The synergetic mechanism between nickel and palladium was investigated in detail, in which the atomic-scale cluster of Ni/Pd could be formed. The real active centre was the crucial factor that made it easy for the initial oxidation step to occur. The absorption and adsorption on the Ni/Pd clusters in the bimetallic Pd–Ni core–shell nanoparticles as effective catalysts for the Suzuki reaction surface for different substrates and products, as calculated by DFT, was also an important factor. This indicated that the synergy between the active site and the doped second metal made it easy for the initial oxidation step and *trans*-metallation to occur, by which the proper design of the catalyst, including the ligand, selection of the support, the suitable combination of metals and elucidation of the catalytic mechanism could be achieved.

## Conflicts of interest

There are no conflicts to declare.

## Supplementary Material

RA-010-D0RA02195D-s001
