# Supplementary material for: Terpyridine-based Pd(ii)/Ni(ii) organometallic framework nano-sheets supported on graphene oxide—investigating the fabrication, tuning of catalytic properties and synergetic effects
Source: RSC Adv. 2020 Jun 17;10(39):23080–90. doi: 10.1039/d0ra02195d (PMC9054763; doi:10.1039/d0ra02195d)
Supplement: RA-010-D0RA02195D-s001 [file RA-010-D0RA02195D-s001.pdf]

Electronic Supplementary Material (ESI) for RSC Advances.
This journal is © The Royal Society of Chemistry 2020

Terpyridine Based Pd(II)/Ni(II) Organometallic Framework Nano-Sheet Supported on Graphene Oxide—Investigating on Fabrication, Turning on Catalytic Properties and Synergetic

Ruirui Ren [a†],Sa Bi,[a†] Linhong Wang,[a] Wuduo Zhao,[a] Donghui Wei,[a] Tiesheng Li,[a*] Wenjian Xu,[a] Minghua Liu,[b,c*]Yangjie Wu[a*]

[a]*College of Chemistry, Zhengzhou University, Zhengzhou,450001, P. R. China.*

[b] *Henan Institute of Advanced Technology, Zhengzhou University, Zhengzhou 450001, P. R. China.*

[c]*Beijing National Laboratory for Molecular Science, Institute of Chemistry, Chinese Academy of Sciences, Zhongguancun North First Street 2, Beijing 100190, P. R China.*

[†]*Contributed equally to this work*

1. Experimental section

1.1 General methods

Chemicals were obtained from commercial sources. Solvents were distilled using appropriate drying agents under nitrogen. X-Ray diffraction (XRD) was performed on a PAN analytical X-Pert PRO instrument. Fourier transform infrared (FTIR) spectroscopy was carried out on a BRUKER TENSOR FTIR spectrometer using KBr pellets. Raman spectra were measured with a Thermo Scientific DXR Raman microscope with an excitation laser wavelength of 532 nm. XPS data were obtained using an ESCALab220i-XL electron spectrometer from VG Scientific with 300 W Al Kα radiation. Scanning electron microscopy (SEM) images were recorded using a Hitachi S-4800 system. Transmission electron microscopy (TEM) images were recorded using a JEM-2100F transmission electron microscope operating at 200 kV. The Pd and Ni content in the catalysts before and after the cross-coupling reactions was measured by inductively coupled plasma atomic emission spectroscopy (ICP-AES) with an ICAP 6000 Series (Thermo Scientific). The sample treatments were as

follows: the sample was broken down with nitrolysis, and then residual solid was dissolved with 2 M hydrochloric acid and transferred into a 10 mL volumetric flask to fix its quantity in water. 1H NMR and[13] C NMR spectra were recorded on a Bruker Advance III 400 MHz spectrometer in $CDCl_3$ with tetramethylsilane as an internal standard. A Vertex 70 V spectrometer (Bruker Optik, Ettlingen, Germany) at 293 K, with a spectral resolution of 4 cm^{-1} and a scanner velocity of 10 kHz was used for monitoring the coupling reaction of 4-bromotoluene with phenylboronic acid. QCM data were recorded on a QCM-2CA(Henan Kerui science and Tech Cop).

1.2. General precedure

1.2.1 Synthesis of functionalized GO with imine ligand, ***GO@Tpy-Ni/Pd***

The aryl imine ligand, functionalized **GO** with aryl imine ligand (**GO@Tpy**) and **GO@Tpy-Ni/Pd** monolayer were prepared according to the literature reports.[27k] **GO@Tpy** (1.1g) is dissolved in 30 mL of EtOH (anhydrous) and sonicated for 0.5 h. Then, a mixture with certain atom ratio of **Ni** to **Pd** was added at 40 °C for 12 h. **GO@Tpy-Ni/Pd** is filtrated, washed with certain solvents and dried in vacuum at 40 °C for 24 h. The real atomic ratios in the as-prepared **GO@Tpy-Ni/Pd*x*** are further determined by ICP–AES. **SiO_2@Tpy-Ni/Pd** is synthesized with similar way for **GO@Tpy-Ni/Pd**.

1.2.2 Procedure for Suzuki reaction and recycling

Si@Typ-Pd/Ni (2.5cm×1cm×0.1cm), base, and reactant were added to a 10 mL round-bottom flask with 4 mL solvent. The reaction was carried out in an oil bath at 80 °C or 100°C for a certain time. The catalysts were separated from the reaction mixture. For the recycling experiments, the reactions were carried out under the above conditions. After each run, the used catalyst was recovered from the reaction mixture

and reused in sequential runs after washing with ethyl acetate, methyl alcohol, and water three times.

1.2.3 ReactIR dynamic analysis

ReactIR analysis are carried out as report. [36] A 10 mL two-neck flask with a magnetic stirrer bar was equipped with the ReactIR probe to monitor the reaction in a 70 °C oil bath. 4 mL solvent, 1.0 mmol reactant, and 2.0 mmol K_2CO_3 were added to the two-neck flask and dispersed by rapid ultrasonic processing. After that, a background spectrum was recorded. Then scans were performed in the time resolved spectroscopy (TRS) mode with 60 s intervals for 30 min.

2. Characterization of GO@Tpy-Ni/Pd monolayer

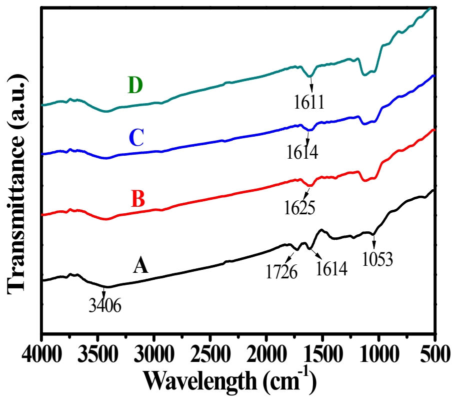

Figure S1 FT-IR spectra of (A) **GO**, (B) **GO@APTES**, (C) **GO@Tpy** and (D) **GO@Tpy-Pd/Ni**.

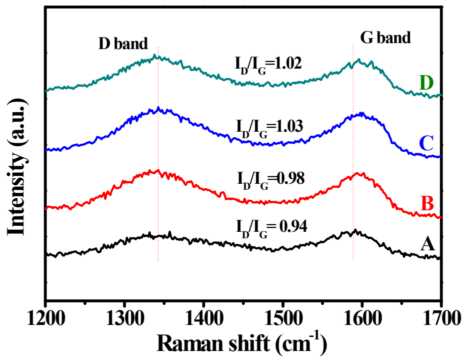

Figure S2 Raman spectra of (A) **GO**, (B) **GO@APTES**, (C) **GO@Tpy** and (D) **GO@Tpy-Pd/Ni**.

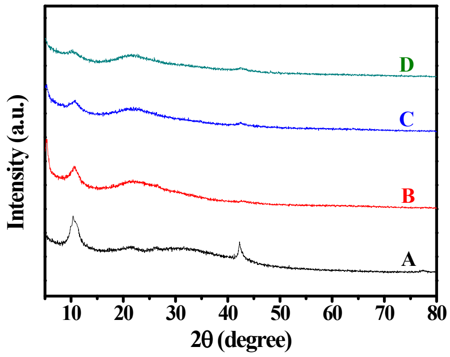

Figure S3 XRD patterns of the self-assembly process of **GO@Tpy-Pd/Ni**. (A) **GO**, (B) **GO@APTES**, (C) **GO@Tpy** and (D) **GO@Tpy-Pd/Ni**.

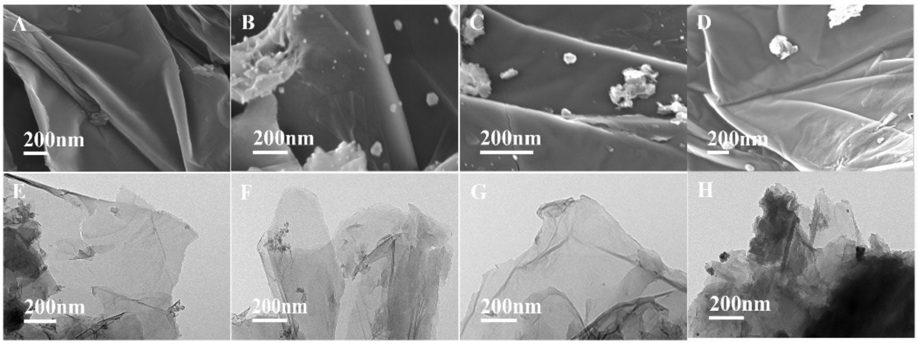

Figure S4 SEM and TEM images of the assembly process of **GO@Tpy-Pd/Ni**. (A, E) **GO**, (B, F) **GO@APTES**, (C, G) **GO@APTES-Tpy**, (D, H) **GO@Tpy-Pd/Ni**.

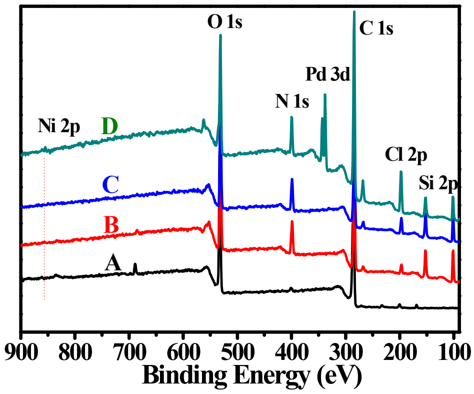

Figure S5 XPS of the self-assembly process of **GO@Tpy-Pd/Ni**. (A) **GO**, (B) **GO@APTES**, (C) **GO@Tpy** and (D) **GO@Tpy-Pd/Ni**.

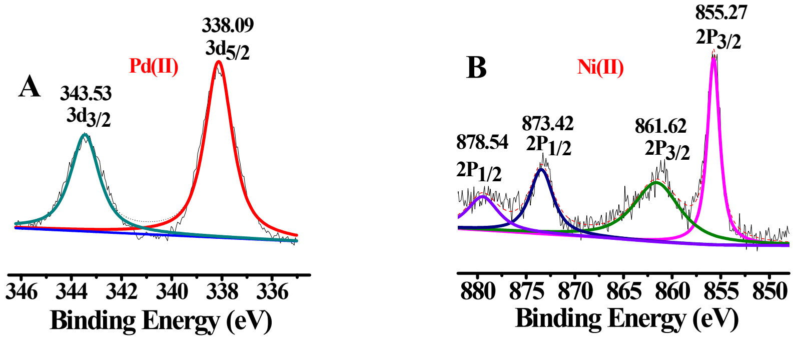

Figure S6 High resolution XPS of (A):Pd 3p，(B):Ni 2p.

3. Investigation on catalytic properties of GO-Tpy-Pd/Ni monolayer

Table S1 The catalytic properties of **GO@Tpy-Pd/Ni** for Suzuki coupling reaction

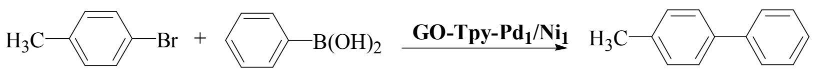

**Entry**	**Base**	**Solvent**	**T(℃)**	**Time (h)**	**Yield (%)**[b]
**1**	K_2CO_3	MeOH	80	12	86
**2**	K_2CO_3	EtOH	80	12	88
**3**	K_2CO_3	EA	80	12	11
**4**	K_2CO_3	DMF	80	12	trace
**5**	K_2CO_3	H_2O	80	12	52
**6**	K_2CO_3	H_2O: EtOH=1:1	80	12	86
**7**	K_2CO_3	H_2O: EtOH=1:3	80	12	94
**8**	K_2CO_3	H_2O: EtOH=1:5	80	12	76
**9**	K_3PO_4	H_2O: EtOH=1:3	80	12	65
**10**	Na_2CO_3	H_2O: EtOH=1:3	80	12	74
**11**	NaOH	H_2O: EtOH=1:3	80	12	45
**12**	K_2CO_3	H_2O: EtOH=1:3	35	12	94
**13**	K_2CO_3	H_2O: EtOH=1:3	35	10	93
**14**	K_2CO_3	H_2O: EtOH=1:3	35	8	94
**15**	K_2CO_3	H_2O: EtOH=1:3	35	6	82
**16**	K_2CO_3	H_2O: EtOH=1:3	35	8[c]	96
**17**	K_2CO_3	H_2O: EtOH=1:3	35	8[d]	96
**18**	K_2CO_3	H_2O: EtOH=1:3	35	8[e]	93
**19**	K_2CO_3	H_2O: EtOH=1:3	35	8[f]	51

[a.]Reaction condition: 4-bromotoluene (0.1 mmol), of PhB(OH)2 (0.15mmol), base(0.2mmol), **GO@Tpy-Pd$_1$/Ni$_1$**: 3mg, solvent (5.0 mL) at 80 °C, [b.]isolated yield.

[c.]**GO@Tpy-Pd$_1$/Ni$_1$**: 2mg,

[d.]**GO@Tpy-Pd$_1$/Ni$_1$**: 1mg.

[e.]$PhB(OH)_2$ (0.35 mmol), 4-bromotoluene (0.3mmol), Base (0.6 mmol), **GO@Tpy-Pd$_1$/Ni$_1$**: 1mg.

[f.]$PhB(OH)_2$ (0.55mmol), 4-bromotoluene (0.5mmol), Base (1 mmol), **GO@Tpy-Pd$_1$/Ni$_1$**: 1 mg.

Table S2 Catalytic activity of **GO@Tpy-Pd/Ni$_x$** for Suzuki–Miyaura reaction.[a]

Catalyst	Pd loading (mol. g^{-1})	Ni loading (mol. g^{-1})	Pd\Ni	Yield/%[b]	TON
**GO-Tpy-Pd**	7.11×10^{-5}	0	-	91	3839
**GO-Tpy-Pd$_1$/Ni$_1$**	6.14×10^{-5}	6.83×10^{-6}	1：0.11	93	4544
**GO-Tpy-Pd$_1$/Ni$_{10}$**	1.82×10^{-5}	2.93×10^{-5}	1：1.6	96	15824
**GO-Tpy-Pd$_1$/Ni$_{20}$**	8.65×10^{-6}	3.11×10^{-5}	1：3.6	41	14219
**GO-Tpy-Ni**	0.00	3.28×10^{-5}	0: 1.0	17	1555

[a] Reaction condition: $PhB(OH)_2$ (0.35 mmol), 4-bromotoluene (0.3mmol), Base (0.6 mmol), **GO@Tpy-Pd/Ni** :1 mg. solvent (5.0 mL) at 35℃ for 8h. [b] isolated yield

Table S3 Suzuki–Miyaura reaction of aryl halides with different arylboronic acids

Entry	Ar–X	Ar'–B(OH)$_2$	Product	Yield (%)[b]	TON	TOF（h^{-1}）
1	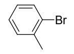	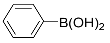	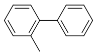	81	13351	1669
2	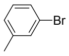	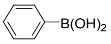	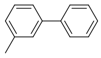	90	14835	1854
3	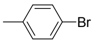	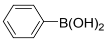	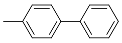	93	15329	1916
4	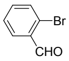	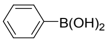	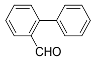	90	14835	1854
5	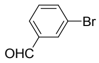	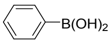	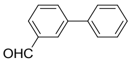	95	15659	1957
6	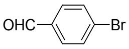	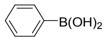	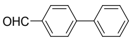	98	16154	2019
7	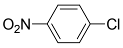	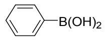	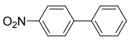	23	3791	473
8	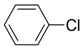	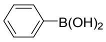	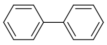	12	1978	274
9	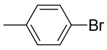	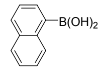	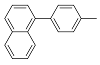	93	15329	1916
10	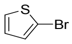	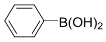	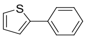	37	6099	762

Table 4 Comparison of the results for the Suzuki reaction using the **GO@Tpy-Pd$_1$/Ni$_{10}$** with that reported Ni or Pd-based catalysts

Entry	catalyst	Pd/Ni loading (mol%)	Reaction condition	Yield (%)	TOF	Ref.
1	**GO@Tpy-Pd$_1$/Ni$_{10}$**	1.82×10^{-5}Pd 2.93×10^{-5} Ni	EtOH/H_2O, 35 °C	96	1978 (based on Pd)	**This work**
2	Pd1Ni2	2.0 Pd 4.0 Ni	DMF, 140 °C	91	5 (based on Pd)	7
3	Ni0.9Pd0.1	0.2 Pd 1.8 Ni	EtOH/H_2O, 50 °C	71	15 (based on Pd)	8

**4**	Pd-Ni alloy	2.0 Pd<br>2.0 Ni	1,4-dioxane, 100 °C	92	3.8<br>Based on Pd	61
**5**	Ni0.20Pd0.05 /CB	0.1 Pd<br>0.4 Ni	EtOH/H_2O,<br>30 °C	88	1333<br>(based on Pd)	63
**6**	NPFNP-2	0.3 pd<br>0.24 Ni	EtOH/H_2O,<br>50 °C	93	270<br>(based on Pd)	64
**7**	Pd50Ni50/MWCNTs	0.1 Pd<br>0.1 Ni	H2O, 120 °C, TBAB	>99	1000<br>(based on Pd)	65
**8**	Pd1Ni4/CNF	1.25×10^{-3} Pd<br>5.1×10^{-3} Ni	EtOH/H_2O,<br>80 °C	98.74	263<br>Based on Pd	67
**9**	Ni0.99Pd0.01 alloy	0.02 Pd<br>1.98 Ni	EtOH/H_2O,<br>50 °C	72	3600<br>(based on Pd)	69
**10**	G–Ni/Pd (Ni/Pd = 3/2)	0.91 Pd<br>1.37 Ni	DMF/H_2O, 110 °C	78	171<br>(based on Pd)	79

Table S5 Poisoning experiments of **GO@Tpy-Pd/Ni** catalyst.[a]

**Entry**	**poisoning additive**	**Isolated yield (%)[d]**
**1**	—	93
**2[b]**	Hg	19
**3[c]**	Thiophene	11

[a] Reaction condition: $PhB(OH)_2$ (0.35 mmol), Base (0.6 mmol), 4-bromotoluene (0.3 mmol), **GO-Tpy-Pd_1/Ni_{10}**: 1 mg, solvent (5.0 mL) at 35 ℃ for 8 h. [b] One drop of Hg. [c] 0.5 equiv of thiophene (per metal atom).[d] Isolated yield.

Table S6 EB values between active sites and substrates

Active site and Substrate	ΔG（a.u）	BE（kcal/mol）
A	-1368.4	
B	-1411.0	
C	-908.3	
S1	-408.2	
S2	-284.0	
CP	-502.5	
A+ S1	-1776.6	7.7
A+ S2	-1652.5	3.8
B+ S1	-1819.2	7.8
B+ S2	-1695.1	3.2

C+ S1	-1316.5	1.4
C+ S2	-1192.4	4.7
A+ CP	-1870.9	10.7
B+ CP	-1913.5	12.6
C+ CP	-1410.8	11.2

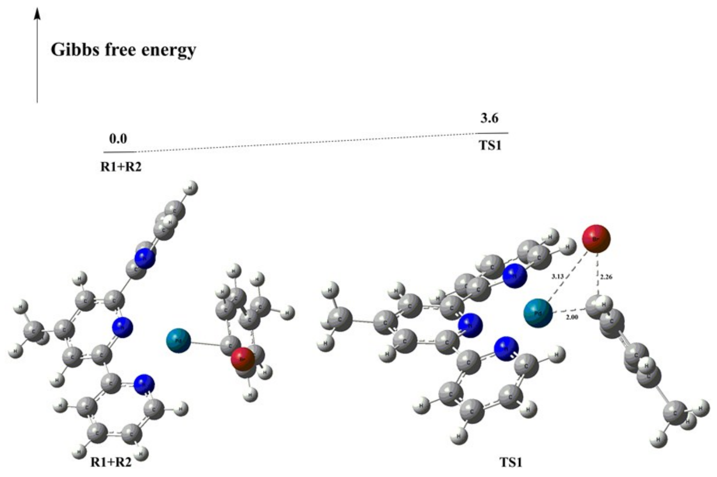

Figure S7. The Gibbs free energy barrier of the oxidative insertion catalyzed by Pd(0) (energy in kcal/mol, distance in Å)

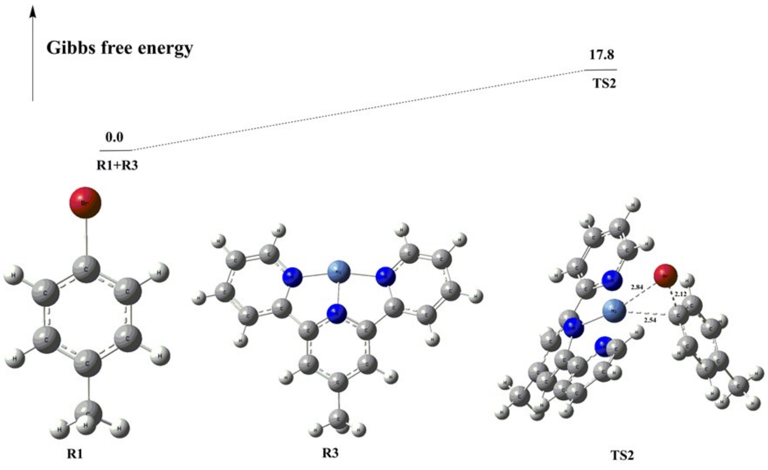

Figure S8. The Gibbs free energy barrier of the oxidative insertion catalyzed by Ni(0) (energy in kcal/mol, distance in Å)

Computational Methods:

The DFT calculations were performed using the Gaussian 09 program[1]. The geometries were fully optimized using B3LYP method[2, 3]. Basis set 6-31+G(d) was employed for H, C, and N atoms, while LANL2DZ was used for Pd、Ni and Br

atoms[4]. Then, frequency calculations at the same level of theory were carried out to identify all of the stationary points as minima (zero imaginary frequency) or transition state (only one frequency), and to provide corrections for free energies.

References

[1]G.W. Trucks, M.J. Frisch, H.B. Schlegel, G.E. Scuseria, M.A. Robb, J.R. Cheeseman, G. Scalmani, V. Barone, B. Mennucci, G.A. Petersson, H. Nakatsuji, M. Caricato, X. Li, H.P. Hratchian, A.F. Izmaylov, J. Bloino, G. Zheng, J.L. Sonnenberg, M. Hada, M. Ehara, K. Toyota, R. Fukuda, J. Hasegawa, M. Ishida, T. Nakajima, Y. Honda, O. Kitao, H. Nakai, T. Vreven, J.A. Montgomery, Jr, J.E. Peralta, F. Ogliaro, M. Bearpark, J.J. Heyd, E. Brothers, K.N. Kudin, V.N. Staroverov, T. Keith, R. Kobayashi, J. Normand, K. Raghavachari, A. Rendell, J.C. Burant, S.S. Iyengar, J. Tomasi, M. Cossi, N. Rega, J.M. Millam, M. Klene, J.E. Knox, J.B. Cross, V. Bakken, C. Adamo, J. Jaramillo, R. Gomperts, R.E. Stratmann, O. Yazyev, A.J. Austin, R. Cammi, C. Pomelli, J.W. Ochterski, R.L. Martin, K. Morokuma, V.G. Zakrzewski, G.A. Voth, P. Salvador, J.J. Dannenberg, S. Dapprich, A.D. Daniels, O. Farkas, J.B. Foresman, J.V. Ortiz, J. Cioslowski, D.J. Fox , Revision C.01, (2010).

[2] A.D. Becke, J. Chem. Phys., 1993, 98, 5648.

[3] C.T. Lee, W.T. Yang, R.G. Parr, Phys. Rev. B., 1988, 37, 785.

[4] P. J. Hay, W. R. Wadt, J. Chem. Phys. 1985, 82, 299.

Geometrical Coordinates of the Listed Complexes

R1

s-2
Zero-point correction=0.117774
Thermal correction to Energy= 0.125429
Thermal correction to Enthalpy= 0.126374
Thermal correction to Gibbs Free Energy= 0.083865
Sum of electronic and zero-point Energies= -284.012700
Sum of electronic and thermal Energies= -284.005044
Sum of electronic and thermal Enthalpies= -284.004100
Sum of electronic and thermal Free Energies= -284.046608

Cartesian coordinates

C	0.312316	-0.000033	-0.000471
C	-0.366751	1.213842	-0.004435
C	-1.762742	1.201116	-0.010294
C	-2.483896	-0.000027	-0.010151
C	-1.762764	-1.201143	-0.010306
C	-0.366737	-1.213875	-0.004424
H	0.177645	2.151398	-0.006843
H	-2.297185	2.147825	-0.017095
H	-2.297178	-2.147867	-0.017109

H	0.177614	-2.151457	-0.006822
C	-3.994214	0.000038	0.015561
H	-4.372214	0.001290	1.045746
H	-4.403386	0.884838	-0.481378
H	-4.403432	-0.885912	-0.479274
Br	2.284768	0.000011	0.003140

R2

j3-1
Zero-point correction=0.254194
Thermal correction to Energy= 0.270111
Thermal correction to Enthalpy= 0.271055
Thermal correction to Gibbs Free Energy= 0.209988
Sum of electronic and zero-point Energies= -908.300142
Sum of electronic and thermal Energies= -908.284225
Sum of electronic and thermal Enthalpies= -908.283281
Sum of electronic and thermal Free Energies= -908.344348

Cartesian coordinates

C	-3.622132	0.726053	0.633414
C	-2.350942	0.299024	0.228235
C	-3.182800	-1.831372	-0.218980
C	-4.465995	-1.471543	0.175277
C	-4.692723	-0.159732	0.604544
H	-3.751828	1.741068	0.994277
H	-2.970791	-2.832796	-0.579479
H	-5.268948	-2.199838	0.131586
H	-5.680371	0.158338	0.924229
C	-1.170867	1.183573	0.225896
C	-1.198864	2.530482	-0.134879
C	1.170863	1.183562	0.225836
C	0.000007	3.245570	-0.281724
H	-2.147573	3.016107	-0.345946
C	1.198958	2.530368	-0.134922
H	2.147705	3.015906	-0.346066
C	2.350932	0.298922	0.228191
C	3.622088	0.725888	0.633487
C	4.692652	-0.159951	0.604668
H	3.751800	1.740890	0.994378
C	3.182716	-1.831468	-0.219082
C	4.465900	-1.471712	0.175302
H	5.680286	0.158060	0.924451
H	2.970693	-2.832860	-0.579656
H	5.268819	-2.200046	0.131650

N	-2.135142	-0.983787	-0.213052
N	-0.000025	0.544123	0.500337
N	2.135090	-0.983846	-0.213128
Pd	-0.000032	-1.481272	-0.285763
C	0.000317	4.709302	-0.646901
H	-0.889757	4.979714	-1.223033
H	0.011396	5.341749	0.250566
H	0.879934	4.974970	-1.241149

R3

A-Ni
Zero-point correction=0.254762
Thermal correction to Energy= 0.269976
Thermal correction to Enthalpy= 0.270920
Thermal correction to Gibbs Free Energy= 0.212454
Sum of electronic and zero-point Energies= -950.867664
Sum of electronic and thermal Energies= -950.852450
Sum of electronic and thermal Enthalpies= -950.851505
Sum of electronic and thermal Free Energies= -950.909972

Cartesian coordinates

C	-3.651200	0.449404	-0.285832
C	-2.297797	0.123390	-0.115420
C	-2.866384	-2.129705	0.216172
C	-4.212149	-1.865982	0.030686
C	-4.618295	-0.539948	-0.206308
H	-3.924155	1.475753	-0.509063
H	-2.517824	-3.132259	0.443848
H	-4.933383	-2.673352	0.097133
H	-5.667714	-0.297903	-0.341504
C	-1.185109	1.052585	-0.220549
C	-1.202397	2.420765	0.030910
C	1.185097	1.052532	-0.220751
C	0.000092	3.145519	0.107046
H	-2.146809	2.925475	0.216469
C	1.202666	2.420591	0.030555
H	2.147198	2.925185	0.215874
C	2.297775	0.123147	-0.115631
C	3.651141	0.449008	-0.286252
C	4.618187	-0.540417	-0.206458
H	3.924167	1.475274	-0.509770
C	2.866144	-2.129853	0.216658
C	4.211941	-1.866292	0.031046

H	5.667619	-0.298500	-0.341776
H	2.517506	-3.132295	0.444690
H	4.933105	-2.673704	0.097755
N	-1.906989	-1.175197	0.173851
N	-0.000046	0.402098	-0.496047
N	1.906837	-1.175284	0.173930
C	0.000567	4.623707	0.411291
H	-0.890598	5.113801	0.006555
H	0.014460	4.818655	1.492126
H	0.878519	5.118150	-0.016625
Ni	-0.000085	-1.383440	0.115685

TS1

pd-ts2
Zero-point correction=0.372536
Thermal correction to Energy=0.398658
Thermal correction to Enthalpy= 0.399602
Thermal correction to Gibbs Free Energy= 0.312140
Sum of electronic and zero-point Energies= -1192.331191
Sum of electronic and thermal Energies= -1192.305069
Sum of electronic and thermal Enthalpies= -1192.304125
Sum of electronic and thermal Free Energies= -1192.391587

Cartesian coordinates

C	-1.840411	3.969943	0.188532
C	-1.460114	2.655364	-0.121919
C	0.684682	3.344213	-0.620099
C	0.381698	4.680115	-0.362533
C	-0.908776	4.995550	0.060845
H	-2.840777	4.181470	0.549377
H	1.685628	3.043996	-0.914299
H	1.142536	5.444696	-0.480601
H	-1.182685	6.018875	0.299779
N	-0.205279	2.351527	-0.509394
C	-2.420913	1.525642	-0.022692
C	-3.798717	1.728295	-0.168675
C	-4.681576	0.653047	-0.055074
H	-4.180492	2.716968	-0.399942
C	-2.740673	-0.742562	0.339964
C	-4.126497	-0.600421	0.214627
H	-4.768898	-1.471460	0.290811
N	-1.901420	0.305184	0.213998
C	-2.124018	-2.072197	0.591144
C	-2.803656	-3.086487	1.281806

C	-2.187284	-4.324642	1.441085
H	-3.784926	-2.902311	1.705556
C	-0.300650	-3.447632	0.252494
C	-0.913867	-4.516663	0.905781
H	-2.691646	-5.123043	1.977413
H	0.689760	-3.538345	-0.186023
H	-0.400950	-5.468893	0.993189
N	-0.882308	-2.251424	0.104441
Pd	0.360289	-0.063908	-0.119964
C	3.420039	-0.300707	1.936522
C	4.113350	0.866568	1.605513
C	3.935372	1.397219	0.312348
C	3.086427	0.803077	-0.612777
C	2.341360	-0.347614	-0.246436
C	2.570619	-0.937497	1.021900
H	3.564842	-0.748535	2.917619
H	4.490204	2.288145	0.022627
H	3.003600	1.191182	-1.622444
H	2.132159	-1.898846	1.263419
Br	2.191986	-1.801173	-1.965412
C	-6.164942	0.825067	-0.262887
H	-6.440058	0.598655	-1.300156
H	-6.738395	0.151017	0.380198
H	-6.482088	1.850782	-0.055606
C	5.036715	1.539622	2.593102
H	5.997526	1.801166	2.135006
H	4.604116	2.471303	2.981534
H	5.241775	0.893206	3.451590

TS2

qst3-a3
Zero-point correction= 0.372497
Thermal correction to Energy= 0.397907
Thermal correction to Enthalpy= 0.398851
Thermal correction to Gibbs Free Energy= 0.314821
Sum of electronic and zero-point Energies= -1234.870548
Sum of electronic and thermal Energies= -1234.845138
Sum of electronic and thermal Enthalpies= -1234.844194
Sum of electronic and thermal Free Energies= -1234.928224

Cartesian coordinates

C	-0.529627	2.948317	-1.164276
C	-0.137865	4.270498	-1.290329

C	1.167285	4.631403	-0.912445
C	2.022728	3.640345	-0.458984
C	1.569444	2.316688	-0.356172
N	0.268945	1.974132	-0.671528
H	1.508230	5.658063	-1.001888
H	-1.526437	2.634857	-1.454785
H	-0.840649	5.005887	-1.667131
H	3.054170	3.867828	-0.210525
C	2.388695	1.183100	0.022698
C	3.531364	1.200520	0.814829
C	2.388698	-1.183109	0.022713
C	4.150912	0.000004	1.201491
H	3.929757	2.149102	1.164569
C	1.569447	-2.316706	-0.356123
C	2.022736	-3.640361	-0.458932
C	1.167277	-4.631431	-0.912337
H	3.054189	-3.867832	-0.210510
C	-0.529661	-2.948364	-1.164107
H	1.508226	-5.658090	-1.001783
H	-1.526488	-2.634914	-1.454567
C	3.531373	-1.200516	0.814837
C	-0.137898	-4.270546	-1.290154
N	1.873870	-0.000011	-0.461450
N	0.268936	-1.974163	-0.671437
H	3.929775	-2.149091	1.164586
H	-0.840697	-5.005949	-1.666900
C	5.366660	0.000006	2.094659
H	5.092363	0.001099	3.158297
H	5.987002	-0.885409	1.922746
H	5.988187	0.884309	1.921258
C	-2.298512	-0.000058	0.373957
C	-2.477749	1.212410	1.045568
C	-2.683808	1.198937	2.423909
C	-2.779316	0.000019	3.144235
C	-2.683981	-1.198870	2.423977
C	-2.477928	-1.212433	1.045571
H	-2.429508	2.156628	0.518642
H	-2.784687	2.148276	2.945020
H	-2.784966	-2.148168	2.945129
H	-2.429805	-2.156696	0.518712
C	-2.980519	0.000243	4.640088
H	-2.020069	0.006832	5.171779
H	-3.538111	0.882408	4.969842
H	-3.527078	-0.888106	4.971570

Br	-2.670638	-0.000030	-1.712000
Ni	-0.006138	-0.000010	-0.727900

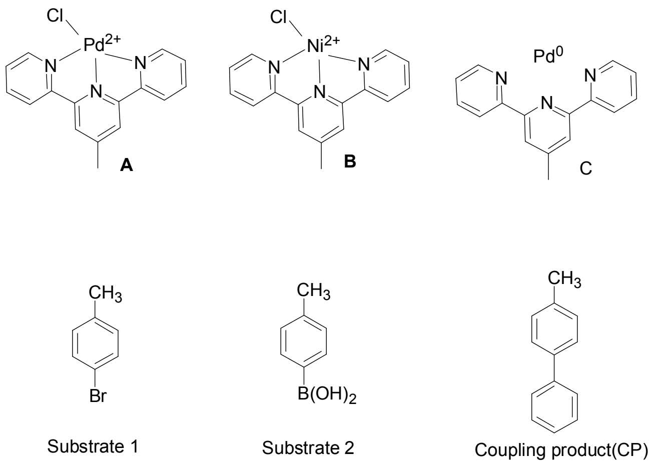

Figure S9 Absorption of active sites on the surface for different substrates and coupling product(CP)

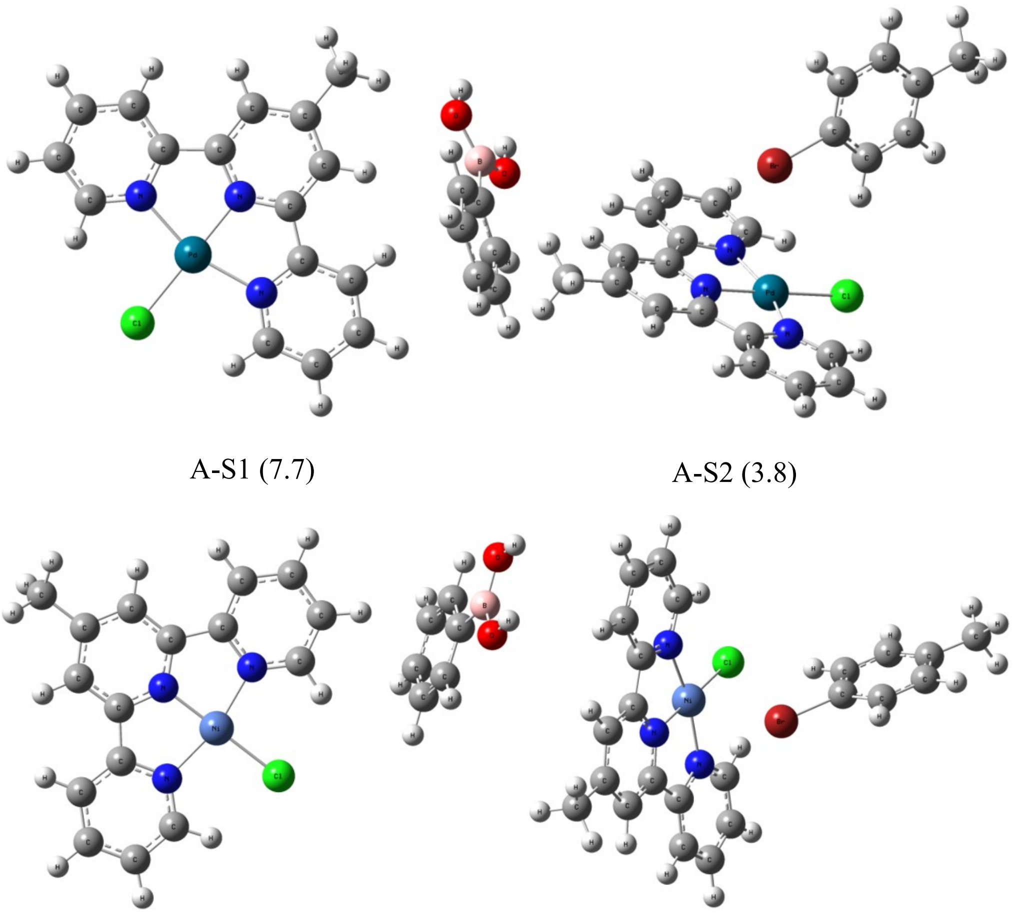

A-S1 (7.7) A-S2 (3.8)

B-S1 (7.8) B-S2 (3.2)

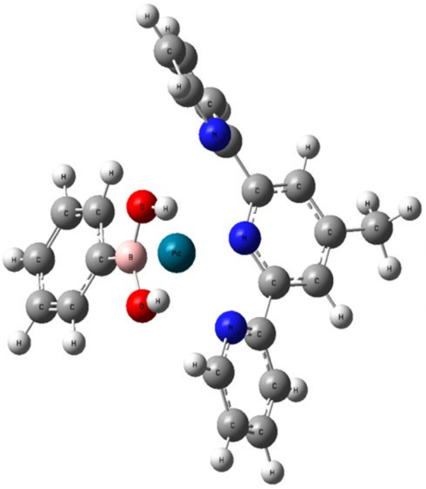

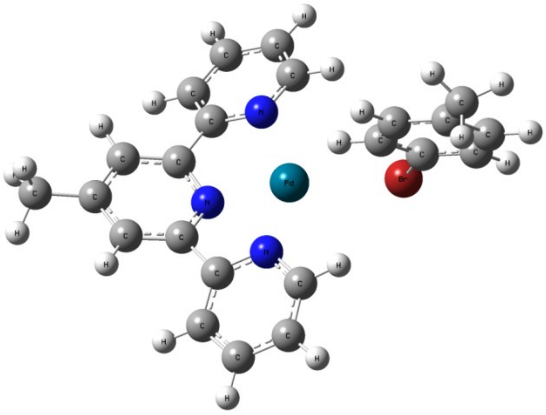

C-S1(1.4)

C-S2(4.7)

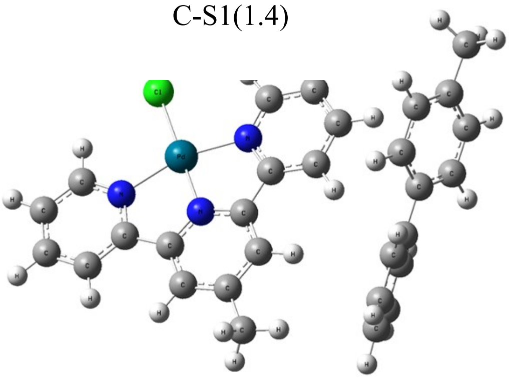

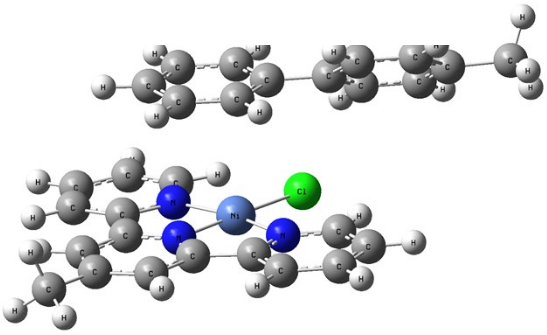

A-S3(10.7)

C-S3(11.2)

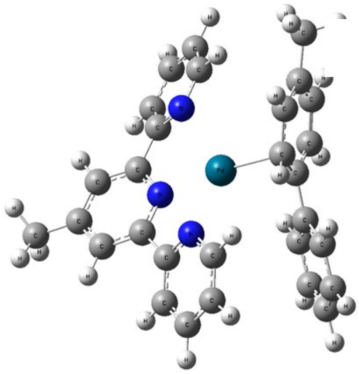

B-S3(12.6)

Figure S10 Calaculated BE for different active sites with different subatrates

Computational Methods:

The DFT calculations were performed using the Gaussian 09 program[1]. The geometries were fully optimized using B3LYP method[2, 3]. Basis set 6-31G(d, p)

was employed for H, C, N, O, B and Cl atoms, while LANL2DZ was used for Pd, Ni and Br atoms.[4] Then, frequency calculations at the same level of theory were carried out to identify all of the stationary points as minima (zero imaginary frequency)and to provide corrections for free energies.

References

[1]G.W. Trucks, M.J. Frisch, H.B. Schlegel, G.E. Scuseria, M.A. Robb, J.R. Cheeseman, G. Scalmani, V. Barone, B. Mennucci, G.A. Petersson, H. Nakatsuji, M. Caricato, X. Li, H.P. Hratchian, A.F. Izmaylov, J. Bloino, G. Zheng, J.L. Sonnenberg, M. Hada, M. Ehara, K. Toyota, R. Fukuda, J. Hasegawa, M. Ishida, T. Nakajima, Y. Honda, O. Kitao, H. Nakai, T. Vreven, J.A. Montgomery, Jr, J.E. Peralta, F. Ogliaro, M. Bearpark, J.J. Heyd, E. Brothers, K.N. Kudin, V.N. Staroverov, T. Keith, R. Kobayashi, J. Normand, K. Raghavachari, A. Rendell, J.C. Burant, S.S. Iyengar, J. Tomasi, M. Cossi, N. Rega, J.M. Millam, M. Klene, J.E. Knox, J.B. Cross, V. Bakken, C. Adamo, J. Jaramillo, R. Gomperts, R.E. Stratmann, O. Yazyev, A.J. Austin, R. Cammi, C. Pomelli, J.W. Ochterski, R.L. Martin, K. Morokuma, V.G. Zakrzewski, G.A. Voth, P. Salvador, J.J. Dannenberg, S. Dapprich, A.D. Daniels, O. Farkas, J.B. Foresman, J.V. Ortiz, J. Cioslowski, D.J. Fox , Revision C.01, (2010).

[2] A.D. Becke, J. Chem. Phys., 1993, 98, 5648.

[3] C.T. Lee, W.T. Yang, R.G. Parr, Phys. Rev. B., 1988, 37,785.

[4]P. J. Hay, W. R. Wadt, J. Chem. Phys. 1985, 82, 299.

Geometrical Coordinates and Vibrational Frequencies of the Listed Compounds

j1-1（A）

Zero-point correction=0.260183
Thermal correction to Energy=0.277867
Thermal correction to Enthalpy=0.278811
Thermal correction to Gibbs Free Energy=0.213368
Sum of electronic and zero-point Energies= -1368.385062
Sum of electronic and thermal Energies= -1368.367378
Sum of electronic and thermal Enthalpies= -1368.366434
Sum of electronic and thermal Free Energies= -1368.431877

Cartesian coordinates

C	-3.679003	1.024162	-0.002937
C	-2.349189	0.613609	-0.001766
C	-3.006233	-1.644323	0.001272
C	-4.355383	-1.286145	-0.000086
C	-4.693332	0.063406	-0.002209

H	-3.925980	2.079418	-0.004284
H	-2.671414	-2.676380	0.002911
H	-5.113514	-2.060904	0.000582
H	-5.733252	0.373059	-0.003198
C	-1.189430	1.538061	-0.002118
C	-1.209153	2.932481	-0.009002
C	1.189663	1.537917	-0.002083
C	0.000234	3.647290	-0.009344
H	-2.149728	3.471604	-0.015925
C	1.209603	2.932242	-0.008946
H	2.150271	3.471235	-0.015825
C	2.349256	0.613241	-0.001739
C	3.679143	1.023558	-0.002920
C	4.693305	0.062628	-0.002221
H	3.926310	2.078771	-0.004257
C	3.005917	-1.644804	0.001255
C	4.355126	-1.286866	-0.000116
H	5.733278	0.372102	-0.003221
H	2.670913	-2.676800	0.002879
H	5.113125	-2.061754	0.000526
N	-2.037094	-0.720914	0.000517
N	0.000033	0.906410	0.001462
N	2.036937	-0.721223	0.000526
Pd	-0.000098	-1.070519	0.001592
Cl	-0.000314	-3.393307	0.001379
C	0.000694	5.152754	0.014286
H	-0.891235	5.560944	-0.466732
H	0.012056	5.513281	1.049962
H	0.882586	5.560352	-0.485570

j1-Ni（B）

Zero-point correction= 0.261298
Thermal correction to Energy=0.278589
Thermal correction to Enthalpy=0.279533
Thermal correction to Gibbs Free Energy= 0.215383
Sum of electronic and zero-point Energies= -1410.977430
Sum of electronic and thermal Energies= -1410.960139
Sum of electronic and thermal Enthalpies= -1410.959195
Sum of electronic and thermal Free Energies= -1411.023344

Cartesian coordinates

C	-3.656565	0.829607	-0.003309
C	-2.313798	0.470077	-0.002132

C	-2.861460	-1.804230	0.001929
C	-4.225274	-1.505698	0.000705
C	-4.627784	-0.174419	-0.001985
H	-3.944100	1.874683	-0.005165
H	-2.486508	-2.821057	0.003938
H	-4.946100	-2.315240	0.001888
H	-5.680987	0.086657	-0.002949
C	-1.180804	1.415193	-0.002751
C	-1.208302	2.808208	-0.008858
C	1.180983	1.415086	-0.002721
C	0.000173	3.525054	-0.008888
H	-2.151899	3.342172	-0.015421
C	1.208654	2.808009	-0.008809
H	2.152327	3.341868	-0.015334
C	2.313829	0.469777	-0.002108
C	3.656654	0.829093	-0.003293
C	4.627716	-0.175081	-0.001995
H	3.944354	1.874125	-0.005139
C	2.861149	-1.804618	0.001912
C	4.225003	-1.506301	0.000678
H	5.680960	0.085833	-0.002970
H	2.486035	-2.821384	0.003908
H	4.945705	-2.315954	0.001838
N	-1.927892	-0.842089	0.000605
N	0.000017	0.773762	0.000279
N	1.927727	-0.842327	0.000611
Cl	-0.000236	-3.265154	0.002731
C	0.000604	5.029867	0.015356
H	-0.891652	5.438525	-0.464669
H	0.012055	5.389115	1.051542
H	0.882772	5.437957	-0.483625
Ni	-0.000092	-1.089031	0.001174

j3-1(C)

Zero-point correction=0.254194
Thermal correction to Energy= 0.270111
Thermal correction to Enthalpy= 0.271055
Thermal correction to Gibbs Free Energy= 0.209988
Sum of electronic and zero-point Energies= -908.300142
Sum of electronic and thermal Energies= -908.284225
Sum of electronic and thermal Enthalpies= -908.283281
Sum of electronic and thermal Free Energies= -908.344348

Cartesian coordinates

C	-3.622132	0.726053	0.633414
C	-2.350942	0.299024	0.228235
C	-3.182800	-1.831372	-0.218980
C	-4.465995	-1.471543	0.175277
C	-4.692723	-0.159732	0.604544
H	-3.751828	1.741068	0.994277
H	-2.970791	-2.832796	-0.579479
H	-5.268948	-2.199838	0.131586
H	-5.680371	0.158338	0.924229
C	-1.170867	1.183573	0.225896
C	-1.198864	2.530482	-0.134879
C	1.170863	1.183562	0.225836
C	0.000007	3.245570	-0.281724
H	-2.147573	3.016107	-0.345946
C	1.198958	2.530368	-0.134922
H	2.147705	3.015906	-0.346066
C	2.350932	0.298922	0.228191
C	3.622088	0.725888	0.633487
C	4.692652	-0.159951	0.604668
H	3.751800	1.740890	0.994378
C	3.182716	-1.831468	-0.219082
C	4.465900	-1.471712	0.175302
H	5.680286	0.158060	0.924451
H	2.970693	-2.832860	-0.579656
H	5.268819	-2.200046	0.131650
N	-2.135142	-0.983787	-0.213052
N	-0.000025	0.544123	0.500337
N	2.135090	-0.983846	-0.213128
Pd	-0.000032	-1.481272	-0.285763
C	0.000317	4.709302	-0.646901
H	-0.889757	4.979714	-1.223033
H	0.011396	5.341749	0.250566
H	0.879934	4.974970	-1.241149

s-1 （s1）

Zero-point correction= 0.125091
Thermal correction to Energy= 0.132994
Thermal correction to Enthalpy= 0.133938
Thermal correction to Gibbs Free Energy= 0.092241
Sum of electronic and zero-point Energies= -408.153798
Sum of electronic and thermal Energies=-408.145895

Sum of electronic and thermal Enthalpies= -408.144951
Sum of electronic and thermal Free Energies= -408.186648

Cartesian coordinates

C	2.644913	0.000052	0.000003
C	1.945414	1.208910	-0.000026
C	0.551142	1.205125	-0.000008
C	-0.173255	-0.000115	-0.000004
C	0.551242	-1.205203	0.000002
C	1.945550	-1.208850	0.000018
H	3.731640	0.000114	0.000010
H	2.487631	2.150539	-0.000065
H	0.009158	2.146405	0.000005
H	0.009421	-2.146586	-0.000035
H	2.487816	-2.150449	0.000046
B	-1.735754	-0.000006	0.000011
O	-2.383914	1.212150	0.000053
H	-3.346409	1.162515	-0.000158
O	-2.384079	-1.212089	0.000004
H	-3.346573	-1.162504	-0.000217

s-2 （S2）

Zero-point correction= 0.117774
Thermal correction to Energy= 0.125429
Thermal correction to Enthalpy= 0.126374
Thermal correction to Gibbs Free Energy= 0.083865
Sum of electronic and zero-point Energies= -284.012700
Sum of electronic and thermal Energies= -284.005044
Sum of electronic and thermal Enthalpies= -284.004100
Sum of electronic and thermal Free Energies= -284.046608

Cartesian coordinates

C	0.312316	-0.000033	-0.000471
C	-0.366751	1.213842	-0.004435
C	-1.762742	1.201116	-0.010294
C	-2.483896	-0.000027	-0.010151
C	-1.762764	-1.201143	-0.010306
C	-0.366737	-1.213875	-0.004424
H	0.177645	2.151398	-0.006843
H	-2.297185	2.147825	-0.017095
H	-2.297178	-2.147867	-0.017109
H	0.177614	-2.151457	-0.006822
C	-3.994214	0.000038	0.015561

H	-4.372214	0.001290	1.045746
H	-4.403386	0.884838	-0.481378
H	-4.403432	-0.885912	-0.479274
Br	2.284768	0.000011	0.003140

s-3（S3）

Zero-point correction= 0.209051
Thermal correction to Energy= 0.219888
Thermal correction to Enthalpy= 0.220832
Thermal correction to Gibbs Free Energy= 0.170321
Sum of electronic and zero-point Energies= -502.433576
Sum of electronic and thermal Energies= -502.422739
Sum of electronic and thermal Enthalpies= -502.421795
Sum of electronic and thermal Free Energies= -502.472306

Cartesian coordinates

C	-0.268204	0.000972	-0.001512
C	-0.995983	1.137515	0.386685
C	-2.388678	1.136729	0.380889
C	-3.113346	0.003188	-0.008685
C	-2.388438	-1.129982	-0.399254
C	-0.995785	-1.133763	-0.395664
C	1.216733	0.000240	0.000091
C	1.941421	1.139659	-0.389049
C	3.335246	1.139279	-0.386056
C	4.037765	-0.002019	0.004017
C	3.332349	-1.142234	0.392075
C	1.938542	-1.140387	0.391107
H	-0.464477	2.024826	0.717823
H	-2.923075	2.031199	0.691939
H	-2.922583	-2.020501	-0.721932
H	-0.463901	-2.018863	-0.732065
H	1.405514	2.023847	-0.721061
H	3.873515	2.029736	-0.698365
H	5.123780	-0.002887	0.005501
H	3.868329	-2.033583	0.705771
H	1.400200	-2.023788	0.721317
C	-4.623146	-0.005761	0.016375
H	-5.032290	-0.678202	-0.743648
H	-5.002853	-0.345733	0.988259
H	-5.033024	0.993327	-0.159647

a+s1-3（a-s1）

Zero-point correction= 0.386469
Thermal correction to Energy= 0.414100
Thermal correction to Enthalpy= 0.415044
Thermal correction to Gibbs Free Energy= 0.324048
Sum of electronic and zero-point Energies= -1776.543830
Sum of electronic and thermal Energies= -1776.516200
Sum of electronic and thermal Enthalpies= -1776.515255
Sum of electronic and thermal Free Energies= -1776.606252

Cartesian coordinates

C	1.799225	-1.611197	-0.072362
C	0.504770	-1.100387	-0.056391
C	-0.399253	-3.258513	-0.291792
C	0.876828	-3.823861	-0.316304
C	1.984625	-2.989959	-0.204116
H	2.656433	-0.953998	0.017463
H	-1.306568	-3.847800	-0.372557
H	0.982100	-4.897796	-0.420174
H	2.991885	-3.392106	-0.214852
C	0.180332	0.339978	0.075799
C	1.072589	1.406433	0.190713
C	-1.672328	1.828405	0.181767
C	0.577161	2.715566	0.310163
H	2.143205	1.235277	0.190368
C	-0.813623	2.919672	0.301314
H	-1.208175	3.925990	0.386758
C	-3.155092	1.838420	0.150113
C	-3.934060	2.988258	0.246163
C	-5.325814	2.879181	0.203290
H	-3.464506	3.959083	0.353490
C	-5.082189	0.502382	-0.025670
C	-5.908591	1.623076	0.065494
H	-5.942063	3.769200	0.277362
H	-5.467035	-0.506296	-0.134151
H	-6.984693	1.498087	0.028246
N	-0.575538	-1.937888	-0.165039
N	-1.142374	0.594489	0.075288
N	-3.748345	0.610912	0.016103
Pd	-2.380127	-0.935949	-0.104349
Cl	-3.840825	-2.733855	-0.314514
C	1.521667	3.877283	0.467345
H	2.508467	3.643959	0.060913
H	1.132725	4.773851	-0.023175
H	1.646114	4.117263	1.530565

C	5.135093	-1.509915	1.937926
C	4.709077	-0.208352	2.214749
C	4.713390	0.754057	1.202706
C	5.135451	0.440196	-0.103811
C	5.564648	-0.876790	-0.358472
C	5.565827	-1.842859	0.649449
H	5.147204	-2.257139	2.726355
H	4.393394	0.057573	3.219582
H	4.411754	1.773049	1.431165
H	5.915877	-1.133567	-1.353580
H	5.922270	-2.847668	0.439031
B	5.175387	1.524042	-1.233522
O	4.545671	2.728754	-0.996402
H	4.669686	3.392590	-1.685422
O	5.815459	1.208241	-2.397565
H	5.831228	1.884415	-3.084151

a+s2-111（a-s2）

Zero-point correction= 0.378291
Thermal correction to Energy= 0.406223
Thermal correction to Enthalpy= 0.407167
Thermal correction to Gibbs Free Energy= 0.310751
Sum of electronic and zero-point Energies= -1652.404915
Sum of electronic and thermal Energies= -1652.376982
Sum of electronic and thermal Enthalpies= -1652.376038
Sum of electronic and thermal Free Energies= -1652.472454

Cartesian coordinates

C	3.168141	0.121243	-1.385993
C	3.404263	0.578294	-0.095876
C	4.731545	0.732240	0.313502
C	5.803297	0.442076	-0.540999
C	5.512425	-0.018413	-1.832440
C	4.196481	-0.182414	-2.269397
H	2.591046	0.809513	0.583397
H	4.928789	1.087435	1.321586
H	6.324952	-0.254898	-2.514361
H	3.986061	-0.538936	-3.271134
C	7.231331	0.645035	-0.094050
H	7.590052	1.644619	-0.368312
H	7.328877	0.550578	0.991018
H	7.904266	-0.080151	-0.560569
Br	1.294278	-0.112223	-2.004313

C	-3.214855	3.026755	-0.876044
C	-2.576205	1.922066	-0.319882
C	-1.316897	3.313440	1.095215
C	-1.926621	4.454353	0.571557
C	-2.886110	4.307224	-0.425278
H	-3.959526	2.894673	-1.652219
H	-0.560742	3.355124	1.872041
H	-1.644434	5.431201	0.947233
H	-3.377351	5.175340	-0.852290
C	-2.837428	0.518352	-0.719116
C	-3.721029	0.060476	-1.696158
C	-2.155087	-1.709340	-0.243384
C	-3.827393	-1.316659	-1.950125
H	-4.323471	0.758552	-2.266221
C	-3.026986	-2.204471	-1.212186
H	-3.089008	-3.269361	-1.405722
C	-1.229396	-2.479640	0.621222
C	-1.103920	-3.865518	0.594243
C	-0.194714	-4.488042	1.452358
H	-1.704655	-4.456207	-0.087379
C	0.402163	-2.325316	2.305584
C	0.567808	-3.711014	2.318716
H	-0.088042	-5.567730	1.438878
H	0.964134	-1.659641	2.952049
H	1.284061	-4.157206	2.999048
N	-1.634810	2.087875	0.660987
N	-2.102959	-0.379744	-0.035849
N	-0.470194	-1.732475	1.481896
Pd	-0.824249	0.298054	1.308828
Cl	0.696959	1.098233	2.882515
C	-4.795157	-1.835223	-2.980255
H	-4.994371	-1.090389	-3.754108
H	-4.419022	-2.742097	-3.460089
H	-5.752391	-2.085656	-2.507247

b+s1-22（b-s1）

Zero-point correction= 0.386979
Thermal correction to Energy= 0.414599
Thermal correction to Enthalpy= 0.415543
Thermal correction to Gibbs Free Energy= 0.321262
Sum of electronic and zero-point Energies= -1819.131773
Sum of electronic and thermal Energies= -1819.104153

Sum of electronic and thermal Enthalpies= -1819.103209
Sum of electronic and thermal Free Energies= -1819.197490

Cartesian coordinates

C	-5.574553	-1.202630	0.255990
C	-4.260041	-0.758108	0.168415
C	-3.452778	-2.952687	0.201902
C	-4.750212	-3.459887	0.291351
C	-5.823579	-2.575506	0.318656
H	-6.392787	-0.491986	0.274921
H	-2.576655	-3.590243	0.175959
H	-4.899191	-4.532578	0.337616
H	-6.842609	-2.941977	0.387376
C	-3.845072	0.655866	0.095010
C	-4.641834	1.798259	0.101257
C	-1.884376	1.964599	-0.057244
C	-4.036764	3.064423	0.022568
H	-5.721109	1.718994	0.167714
C	-2.636486	3.138361	-0.054923
H	-2.149077	4.105288	-0.110352
C	-0.420359	1.806966	-0.128420
C	0.493374	2.849968	-0.215677
C	1.857822	2.553411	-0.276754
H	0.149490	3.877826	-0.236808
C	1.298528	0.220940	-0.159011
C	2.266631	1.224887	-0.248446
H	2.586453	3.354523	-0.346838
H	1.563655	-0.828619	-0.135491
H	3.313438	0.946292	-0.295667
N	-3.211530	-1.635264	0.141945
N	-2.508803	0.776995	0.016851
N	-0.010994	0.501106	-0.101454
Cl	-0.269390	-2.586018	0.013250
C	-4.874928	4.314359	-0.002800
H	-5.136890	4.571433	-1.036334
H	-5.808327	4.181526	0.549383
H	-4.337525	5.165634	0.421625
Ni	-1.472406	-0.770880	0.018496
C	4.362380	-1.324366	-2.130682
C	5.171305	-0.193658	-2.276056
C	5.861211	0.315279	-1.173742
C	5.757652	-0.289137	0.093658
C	4.936129	-1.425729	0.217619
C	4.245584	-1.942127	-0.881060

H	3.835294	-1.731193	-2.989436
H	5.273256	0.280155	-3.248555
H	6.501959	1.184579	-1.292044
H	4.854630	-1.914128	1.184453
H	3.629788	-2.830568	-0.769385
B	6.561338	0.264763	1.320701
O	7.327162	1.385595	1.126732
H	7.840501	1.678387	1.888098
O	6.443518	-0.401262	2.512686
H	6.972047	-0.055630	3.240707

b+s2-333（b-s2）

Zero-point correction= 0.379339
Thermal correction to Energy= 0.406910
Thermal correction to Enthalpy= 0.407854
Thermal correction to Gibbs Free Energy= 0.312681
Sum of electronic and zero-point Energies= -1694.998161
Sum of electronic and thermal Energies= -1694.970589
Sum of electronic and thermal Enthalpies= -1694.969645
Sum of electronic and thermal Free Energies= -1695.064818

Cartesian coordinates

C	3.126610	-0.003806	-1.210623
C	3.471052	-0.004704	0.134457
C	4.829586	-0.008909	0.462182
C	5.824768	-0.010209	-0.524101
C	5.423959	-0.013155	-1.867223
C	4.074530	-0.008993	-2.225840
H	2.715739	-0.005792	0.912907
H	5.113473	-0.013033	1.511327
H	6.175177	-0.020710	-2.652525
H	3.778201	-0.013463	-3.268450
C	7.288142	0.015937	-0.152771
H	7.665075	1.045289	-0.114506
H	7.460198	-0.430791	0.830348
H	7.896350	-0.525184	-0.883222
Br	1.204316	-0.002777	-1.715968
C	-2.216814	-3.653998	-0.055855
C	-1.970105	-2.311943	0.210443
C	-0.405287	-2.863878	1.857085
C	-0.609138	-4.226971	1.635744
C	-1.524695	-4.627218	0.668144

H	-2.935714	-3.938282	-0.815578
H	0.294129	-2.491159	2.596086
H	-0.051345	-4.949461	2.220417
H	-1.702596	-5.679965	0.474598
C	-2.622014	-1.177999	-0.471249
C	-3.576199	-1.204657	-1.486377
C	-2.619032	1.183055	-0.471823
C	-4.067886	0.003960	-2.006687
H	-3.938060	-2.148130	-1.879229
C	-3.572964	1.211730	-1.486870
H	-3.932359	2.155981	-1.880205
C	-1.964194	2.315564	0.209498
C	-2.207425	3.658158	-0.057341
C	-1.512901	4.629866	0.666352
H	-2.925516	3.943988	-0.817250
C	-0.398130	2.864134	1.856086
C	-0.598478	4.227641	1.634226
H	-1.688076	5.682994	0.472393
H	0.300248	2.489874	2.595292
H	-0.038932	4.948935	2.218702
N	-1.067867	-1.928522	1.162829
N	-2.183110	0.001999	-0.002448
N	-1.063038	1.930204	1.162093
Cl	0.618629	-0.000935	2.919890
C	-5.119909	0.005869	-3.083035
H	-5.060966	-0.892079	-3.702509
H	-6.119703	0.031875	-2.632869
H	-5.028262	0.881541	-3.730124
Ni	-0.884557	0.000645	1.332189

c+s1-11（c-s1）

Zero-point correction= 0.379706
Thermal correction to Energy= 0.406692
Thermal correction to Enthalpy= 0.407636
Thermal correction to Gibbs Free Energy= 0.319104
Sum of electronic and zero-point Energies= -1316.468127
Sum of electronic and thermal Energies= -1316.441141
Sum of electronic and thermal Enthalpies= -1316.440196
Sum of electronic and thermal Free Energies= -1316.528729

Cartesian coordinates

C	2.900434	2.821159	-0.591578
C	2.226346	1.860594	0.175644

C	0.997465	3.479282	1.244568
C	1.612170	4.507778	0.526824
C	2.583291	4.166448	-0.413050
H	3.636833	2.516722	-1.328300
H	0.231803	3.701149	1.985310
H	1.332786	5.542005	0.701185
H	3.079950	4.931422	-1.002937
C	2.537288	0.408715	0.039026
C	3.864826	-0.021550	-0.040713
C	1.762414	-1.773230	-0.034323
C	4.154975	-1.388237	-0.102290
H	4.668302	0.708389	-0.021614
C	3.073120	-2.267991	-0.085140
H	3.251542	-3.337878	-0.086886
C	0.588589	-2.685907	-0.008624
C	0.653855	-4.005632	-0.478175
C	-0.470719	-4.819497	-0.380850
H	1.562412	-4.382985	-0.934206
C	-1.632267	-2.964944	0.598103
C	-1.635276	-4.293734	0.180824
H	-0.440080	-5.842442	-0.744015
H	-2.518922	-2.494277	1.014772
H	-2.533660	-4.893689	0.284075
N	1.287721	2.185497	1.078964
N	1.501828	-0.451778	0.018926
N	-0.552742	-2.171858	0.500604
Pd	-0.778905	0.084256	0.146853
C	5.578519	-1.885097	-0.132211
H	6.197219	-1.276327	-0.798941
H	6.030418	-1.833872	0.865712
H	5.633388	-2.924191	-0.467462
C	-2.463212	1.515165	-2.892633
C	-3.359116	0.487975	-2.504688
C	-3.516518	0.165295	-1.173383
C	-2.815323	0.870641	-0.137410
C	-1.897639	1.899589	-0.552313
C	-1.748033	2.205436	-1.935131
H	-2.350025	1.763761	-3.944910
H	-3.929865	-0.042199	-3.263307
H	-4.218196	-0.612589	-0.882877
H	-1.535491	2.615075	0.180131
H	-1.078240	3.011039	-2.227200
B	-3.107552	0.625732	1.366519
O	-3.772853	-0.542574	1.732916

H	-3.977660	-0.589502	2.673350
O	-2.783507	1.603944	2.291680
H	-2.853033	1.318423	3.209205

c+s2-111（c-s2）

Zero-point correction= 0.372073
Thermal correction to Energy= 0.399042
Thermal correction to Enthalpy= 0.399986
Thermal correction to Gibbs Free Energy= 0.307070
Sum of electronic and zero-point Energies= -1192.318523
Sum of electronic and thermal Energies= -1192.291554
Sum of electronic and thermal Enthalpies= -1192.290610
Sum of electronic and thermal Free Energies= -1192.383526

Cartesian coordinates

C	3.501575	-3.152737	0.085966
C	2.653487	-2.040098	0.202167
C	0.783988	-3.413828	0.063017
C	1.562454	-4.558991	-0.040971
C	2.956943	-4.424528	-0.029924
H	4.576582	-3.007044	0.060927
H	-0.300730	-3.475002	0.056564
H	1.088779	-5.532159	-0.119928
H	3.600568	-5.293770	-0.126265
C	3.155853	-0.665610	0.345157
C	4.315488	-0.332081	1.045003
C	2.632575	1.599381	0.048590
C	4.665261	1.012237	1.234965
H	4.932808	-1.116631	1.473819
C	3.782186	1.982124	0.739223
H	3.975733	3.035014	0.924917
C	1.589869	2.539867	-0.386729
C	1.874518	3.840853	-0.829013
C	0.837759	4.681694	-1.210911
H	2.906595	4.169835	-0.893724
C	-0.684701	2.895753	-0.713308
C	-0.475484	4.196557	-1.150292
H	1.043931	5.687929	-1.562987
H	-1.688758	2.486491	-0.645646
H	-1.321059	4.817946	-1.426978
N	1.291839	-2.172765	0.191552
N	2.360091	0.289728	-0.212548

N	0.306107	2.065724	-0.329119
Pd	0.258280	-0.203833	-0.361654
C	5.889186	1.399644	2.027471
H	6.671054	0.637798	1.950093
H	6.305554	2.349741	1.677889
H	5.657492	1.520124	3.093643
C	-3.510737	-0.463130	-0.144553
C	-4.869968	-0.585881	-0.405033
C	-5.773224	-0.202530	0.588992
C	-5.333955	0.297433	1.822126
C	-3.953380	0.399647	2.041326
C	-3.029693	0.022506	1.064435
H	-5.222852	-0.972903	-1.354747
H	-6.839135	-0.297374	0.397063
H	-3.587519	0.777930	2.992784
H	-1.955910	0.096642	1.231316
C	-6.321382	0.738581	2.877068
H	-6.552286	1.806983	2.781284
H	-7.266195	0.193222	2.795019
H	-5.926121	0.581832	3.885160
Br	-2.205068	-0.999151	-1.535770

pd-ben (A-s3)

Zero-point correction= 0.470194
Thermal correction to Energy= 0.500941
Thermal correction to Enthalpy= 0.501885
Thermal correction to Gibbs Free Energy= 0.401776
Sum of electronic and zero-point Energies= -1870.818685
Sum of electronic and thermal Energies= -1870.787938
Sum of electronic and thermal Enthalpies= -1870.786994
Sum of electronic and thermal Free Energies= -1870.887104

Cartesian coordinates

C	-4.986471	-0.164622	-0.099757
C	-4.877296	0.438545	-1.365488
C	-5.286667	1.754010	-1.574719
C	-5.824428	2.523887	-0.532760
C	-5.923530	1.926682	0.732887
C	-5.516201	0.610352	0.946997
C	-4.562094	-1.571790	0.123606
C	-4.750086	-2.550207	-0.870248
C	-4.375753	-3.875422	-0.653892
C	-3.804274	-4.258099	0.563259
C	-3.603760	-3.298605	1.559103

C	-3.975620	-1.970678	1.340046
H	-4.452985	-0.122389	-2.193374
H	-5.188757	2.190497	-2.565526
H	-6.346778	2.492822	1.558829
H	-5.645698	0.168704	1.930637
H	-5.230774	-2.276479	-1.804369
H	-4.552899	-4.616057	-1.428278
H	-3.540292	-5.296515	0.741680
H	-3.170680	-3.584863	2.513504
H	-3.815939	-1.236026	2.124474
C	-6.317398	3.930088	-0.774581
H	-7.365828	3.924677	-1.096580
H	-6.261393	4.538876	0.132395
H	-5.742272	4.429661	-1.559812
C	5.175483	-2.283527	-0.371805
C	4.153833	-1.349174	-0.225931
C	5.716415	0.406891	-0.169494
C	6.777481	-0.487690	-0.314682
C	6.501670	-1.847878	-0.416440
H	4.943673	-3.339303	-0.449920
H	5.857726	1.479297	-0.083976
H	7.793522	-0.111238	-0.345460
H	7.305550	-2.567838	-0.529636
C	2.711871	-1.691346	-0.165682
C	2.131069	-2.955026	-0.238634
C	0.569272	-0.682671	0.061074
C	0.732899	-3.086747	-0.158238
H	2.747057	-3.839824	-0.355626
C	-0.050612	-1.932615	-0.002238
H	-1.128881	-2.021386	0.075436
C	-0.078183	0.641712	0.213741
C	-1.452987	0.832383	0.302395
C	-1.958721	2.128313	0.439443
H	-2.130361	-0.012196	0.262421
C	0.297058	2.955251	0.392673
C	-1.073844	3.201181	0.487350
H	-3.031096	2.281588	0.504335
H	1.040678	3.744764	0.420108
H	-1.424917	4.221345	0.593546
N	4.446238	-0.014557	-0.126619
N	1.911179	-0.616684	-0.022002
N	0.776666	1.712853	0.258800
Pd	2.760149	1.165355	0.086791
Cl	3.762214	3.259497	0.220789

C	0.094907	-4.447361	-0.248825
H	0.552111	-5.140796	0.464327
H	-0.977567	-4.401210	-0.051592
H	0.239543	-4.870206	-1.249563

Ni-ben(B-S3)

Zero-point correction= 0.470812
Thermal correction to Energy= 0.501344
Thermal correction to Enthalpy= 0.502288
Thermal correction to Gibbs Free Energy= 0.403037
Sum of electronic and zero-point Energies= -1913.407760
Sum of electronic and thermal Energies= -1913.377228
Sum of electronic and thermal Enthalpies= -1913.376284
Sum of electronic and thermal Free Energies= -1913.475535

Cartesian coordinates

C	0.752046	-2.794755	-1.865019
C	-0.037491	-1.720165	-1.474366
C	1.609336	-0.194513	-2.126437
C	2.451561	-1.232270	-2.531096
C	2.017685	-2.546403	-2.402479
H	0.390250	-3.809974	-1.750063
H	1.892083	0.847556	-2.205629
H	3.429657	-0.994543	-2.932502
H	2.650314	-3.372233	-2.711005
C	-1.384752	-1.826472	-0.885276
C	-2.129456	-2.974070	-0.616490
C	-3.121105	-0.453053	-0.067734
C	-3.408749	-2.852958	-0.052084
H	-1.729812	-3.956305	-0.841963
C	-3.905156	-1.566726	0.221942
H	-4.894001	-1.449914	0.651153
C	-3.442367	0.974009	0.122735
C	-4.629472	1.462679	0.657264
C	-4.802839	2.843405	0.774764
H	-5.408130	0.779512	0.976326
C	-2.615156	3.140798	-0.174522
C	-3.783772	3.691700	0.353630
H	-5.722502	3.244107	1.188465
H	-1.787421	3.748509	-0.521129
H	-3.878185	4.769184	0.425497
N	0.394424	-0.430392	-1.613629
N	-1.901696	-0.619348	-0.606846

N	-2.446527	1.815650	-0.287298
Cl	0.277278	2.672455	-1.493046
C	-4.227284	-4.073451	0.274265
H	-3.916021	-4.936638	-0.318059
H	-4.108804	-4.336217	1.332361
H	-5.291789	-3.897417	0.098912
Ni	-0.886363	0.892059	-0.993915
C	3.122876	0.037174	1.384975
C	3.334333	1.184807	0.600466
C	4.590129	1.464299	0.063267
C	5.685045	0.618648	0.288958
C	5.472506	-0.526229	1.069702
C	4.220556	-0.811557	1.609331
C	1.789043	-0.268274	1.963577
C	0.929923	0.759924	2.394727
C	-0.310216	0.469684	2.965841
C	-0.724795	-0.855756	3.120995
C	0.113495	-1.888050	2.692382
C	1.351289	-1.596714	2.118274
H	2.507527	1.855909	0.386557
H	4.721400	2.358864	-0.540867
H	6.304512	-1.196139	1.272167
H	4.103195	-1.688128	2.239838
H	1.254945	1.792744	2.319694
H	-0.937864	1.282071	3.322290
H	-1.676651	-1.081780	3.593377
H	-0.189035	-2.924138	2.819519
H	1.990140	-2.409525	1.785572
C	7.053683	0.948317	-0.256095
H	7.628491	1.543712	0.463897
H	7.633660	0.043862	-0.462470
H	6.989697	1.532007	-1.179109

pd0-ben-2 （C-S3）

Zero-point correction= 0.463320
Thermal correction to Energy= 0.493222
Thermal correction to Enthalpy= 0.494166
Thermal correction to Gibbs Free Energy= 0.397817
Sum of electronic and zero-point Energies= -1410.733277
Sum of electronic and thermal Energies= -1410.703375
Sum of electronic and thermal Enthalpies= -1410.702431
Sum of electronic and thermal Free Energies= -1410.798780

Cartesian coordinates

C	-3.304909	2.371678	1.329954
C	-2.340124	1.939398	0.409138
C	-3.724532	0.257588	-0.341453
C	-4.745365	0.627373	0.533638
C	-4.525732	1.704513	1.392892
H	-3.092775	3.199805	1.998213
H	-3.847310	-0.579858	-1.021488
H	-5.684505	0.083171	0.539635
H	-5.286602	2.015017	2.102965
C	-1.029529	2.620817	0.269045
C	-0.902774	4.002271	0.441438
C	1.217512	2.428141	-0.280052
C	0.336054	4.622781	0.251873
H	-1.776359	4.597418	0.688042
C	1.404368	3.806988	-0.126965
H	2.374023	4.246687	-0.337177
C	2.320241	1.541446	-0.722433
C	3.658049	1.802458	-0.392336
C	4.652814	0.950207	-0.861208
H	3.909367	2.645504	0.242593
C	2.929520	-0.338001	-1.913051
C	4.282005	-0.142224	-1.647880
H	5.694123	1.128105	-0.610086
H	2.599709	-1.181039	-2.515699
H	5.021630	-0.829986	-2.044928
N	-2.543974	0.885875	-0.408971
N	0.020687	1.841634	-0.065438
N	1.958201	0.471087	-1.465634
Pd	-0.239969	-0.354090	-0.646572
C	0.504863	6.110041	0.436280
H	-0.384153	6.656993	0.108302
H	0.666146	6.355308	1.493421
H	1.364948	6.487940	-0.123277
C	-0.148684	-2.445929	0.401275
C	-0.298901	-2.490243	-1.026252
C	-1.562849	-2.838158	-1.588448
C	-2.668034	-3.115949	-0.798874
C	-2.523255	-3.034844	0.608436
C	-1.312361	-2.699309	1.185035
C	1.189808	-2.457656	1.058530
C	2.273329	-3.149030	0.487772
C	3.513350	-3.201364	1.125166

C	3.702280	-2.574071	2.357041
C	2.631945	-1.892547	2.943860
C	1.396155	-1.834851	2.304863
H	0.579960	-2.583353	-1.661996
H	-1.642103	-2.919763	-2.670106
H	-3.373775	-3.263352	1.246721
H	-1.220288	-2.696851	2.267473
H	2.134858	-3.672595	-0.453493
H	4.330517	-3.748733	0.662059
H	4.666337	-2.618251	2.855767
H	2.763114	-1.396439	3.902127
H	0.581935	-1.277386	2.758138
C	-3.986490	-3.536946	-1.406347
H	-4.249376	-4.562373	-1.117565
H	-4.813389	-2.896941	-1.073468
H	-3.954411	-3.498533	-2.499168
